# RPRD1A drives lenvatinib resistance in hepatocellular carcinoma via the ITGA5-FAK signaling axis

**DOI:** 10.1186/s43556-026-00459-8

**Published:** 2026-05-22

**Authors:** Xiaomeng Yao, Yunkai Lin, Pingping Chen, Mengyou Xu, Mengmiao Pei, Xinru Fan, Huibo Feng, Xinhao Xing, Jiaqi Guo, Yang Liu, Xinzhu Quan, Yufei Pan, Yexiong Tan, Huabang Zhou, Liwei Dong, Hui Wang, Heping Hu

**Affiliations:** 1 Department of Hepatobiliary Medicine, Eastern Hepatobiliary Surgery Hospital, Second Military Medical University (Naval Medical University), No. 700 Moyu North Road, Jiading, Shanghai, 201800 China; 2 National Center for Liver Cancer, Naval Medical University, No. 366 Qianju Road, Jiading, Shanghai, 201800 China; 3 International Cooperation Laboratory On Signal Transduction, Eastern Hepatobiliary Surgery Hospital, Second Military Medical University (Naval Medical University), No. 225 Changhai Road, Yangpu, Shanghai, 200438 China; 4 Department of Oncology, Eastern Hepatobiliary Surgery Hospital, Second Military Medical University (Naval Medical University), No. 700 Moyu North Road, Jiading, Shanghai, 201800 China; 5 Laboratory of Signaling Regulation and Targeting Therapy of Liver Cancer, the Ministry of Education, No. 225 Changhai Road, Yangpu, Shanghai, 200438 China

**Keywords:** Hepatocellular carcinoma (HCC), Lenvatinib resistance, Regulator of nuclear pre-mRNA domain-containing protein 1A (RPRD1A), Integrin subunit alpha 5 (ITGA5), Focal adhesion kinase (FAK)

## Abstract

Hepatocellular carcinoma (HCC) constitutes a formidable global health challenge. Lenvatinib, a multi-targeted tyrosine kinase inhibitor (TKI), has gradually become a cornerstone therapeutic agent for advanced HCC; however, the emergence of drug resistance severely compromises its clinical efficacy and remains a major obstacle to improving patient outcomes. In this study, we identified key genes associated with lenvatinib resistance, among which regulator of nuclear pre-mRNA domain-containing protein 1A (RPRD1A) emerged as a prominent candidate. Functional assays demonstrated that RPRD1A overexpression significantly reduces the sensitivity of HCC to lenvatinib, whereas its knockdown restores drug responsiveness. Mechanistically, RPRD1A competes with RNA polymerase II-associated protein 2 (RPAP2) for binding to RNA polymerase II (RNA Pol II), thereby derepressing integrin subunit alpha 5 (ITGA5) transcription via a c-JUN-dependent pathway. Upregulated ITGA5 subsequently activates focal adhesion kinase (FAK) signaling, which critically drives lenvatinib resistance. Notably, combined treatment with lenvatinib and either the ITGA5 inhibitor volociximab or the FAK inhibitor defactinib effectively reversed lenvatinib resistance in preclinical models. Furthermore, clinical analyses revealed that elevated expression of RPRD1A and ITGA5 correlates with poor response to lenvatinib in HCC patients, reinforcing their potential as predictive biomarkers for therapeutic resistance. Collectively, our results demonstrate that co-targeting the RPRD1A-ITGA5-FAK signaling axis in combination with lenvatinib represents a promising strategy to improve therapeutic efficacy in HCC.

**Supplementary Information:**

The online version contains supplementary material available at 10.1186/s43556-026-00459-8.

## Introduction

Hepatocellular carcinoma (HCC), the most prevalent primary liver malignancy, accounts for 75% to 85% of all primary liver tumors [1–3]. Ranked as the sixth most prevalent cancer worldwide and the third leading cause of cancer-related mortality, HCC imposes a considerable public health burden [4]. Surgical resection remains the only curative treatment. However, the typically asymptomatic nature of the early stages results in the majority of patients being diagnosed at an advanced disease stage, rendering them ineligible for surgical resection and necessitating reliance on alternative therapeutic modalities [5]. Even among patients undergoing curative resection, tumor recurrence occurs frequently, with rates exceeding 50% at 2 years and 70% at 5 years postoperatively [6, 7]. This recurrence further reduces 5-year survival by 24%, thereby exacerbating the already poor prognosis [6, 8].

Targeted therapy has emerged as a pivotal advancement in the management of advanced HCC. Sorafenib, the first FDA-approved systemic therapy for unresectable HCC, established a landmark in treatment through the SHARP trial, extended median overall survival (OS) to 10.7 months, and objective response rate (ORR) to 2% [9]. However, subsequent clinical trials evaluating other agents, including Sunitinib [10], Brivanib [11], and Erlotinib [12], failed to replicate or surpass this efficacy. Lenvatinib, approved in 2018 as a first-line therapeutic option for unresectable HCC, represented a significant therapeutic breakthrough. The pivotal REFLECT trial demonstrated that lenvatinib achieved comparable OS to sorafenib while yielding superior anti-tumor activity: median OS extended to 13.6 months (vs. 12.3 months with sorafenib; hazard ratio [HR] = 0.92, 95% confidence interval [CI]: 0.79–1.06), and the ORR surged to 24.1% (vs. 9.2% with sorafenib), solidifying its role as a frontline standard [13, 14].

As an oral multi-target tyrosine kinase inhibitor (TKI), lenvatinib exerts its anti-tumor effects through targeting vascular endothelial growth factor receptors (VEGFR1-3) (impeding angiogenesis), and fibroblast growth factor receptors (FGFR1-4) (suppressing proliferation, metastasis, and apoptosis evasion). It also modulates platelet-derived growth factor receptor (PDGFR), stem cell factor receptor (KIT), and rearranged during transfection (RET), further inhibiting tumor survival and progression [15, 16]. However, the clinical efficacy of lenvatinib is constrained by heterogeneous drug sensitivity, with a substantial proportion of patients failing to respond or developing acquired resistance. This underscores the imperative to decipher the molecular mechanisms underlying treatment resistance and develop strategies to enhance therapeutic responsiveness.

Here, we uncover a novel mechanism of lenvatinib resistance, mediated by regulator of nuclear pre-mRNA domain-containing protein 1A (RPRD1A). Mechanistically, RPRD1A positively regulates the expression of integrin subunit alpha 5 (ITGA5) by competitively binding to RNA polymerase II (RNA Pol II) and displaces RNA polymerase II-associated protein 2 (RPAP2) from RNA Pol II. This upregulation of ITGA5 activates the focal adhesion kinase (FAK) signaling pathway, ultimately driving subsequent non-canonical induction of lenvatinib resistance. Notably, targeted inhibition of this RPRD1A-ITGA5-FAK axis restored lenvatinib sensitivity. The ITGA5 inhibitor volociximab and FAK inhibitor defactinib exhibited synergistic anti-tumor effects with lenvatinib both in vitro and in vivo. This study is the first to demonstrate that RPRD1A confers lenvatinib resistance in HCC via a previously unrecognized mechanism by competitively binding to RNA polymerase II and activating the c-JUN/ITGA5/FAK signaling axis. Our findings identify novel biomarkers and therapeutic targets for overcoming this major clinical obstacle.

## Results

### Elevated RPRD1A expression correlates with acquired lenvatinib resistance in the HCC model

**Fig. 1:**
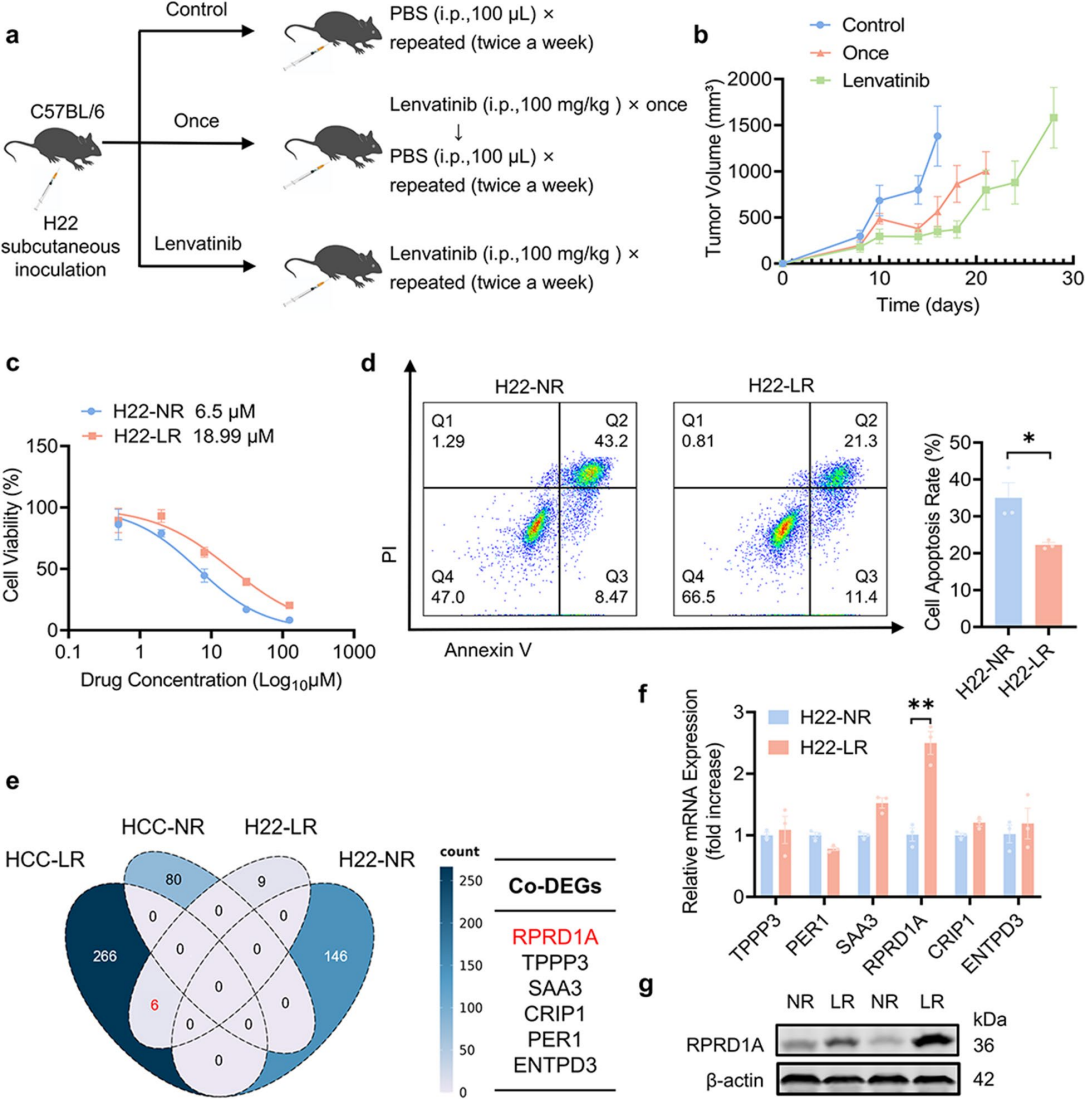
The expression level of RPRD1A is associated with lenvatinib resistance in HCC. **a** Schematic diagram of the acquired lenvatinib resistance murine model. **b** Tumor volumes of the three groups in the xenograft models are shown (n = 5). **c** Dose–response curves of H22-NR and H22-LR cells treated with gradient concentrations of lenvatinib for 48 h, and IC50 values calculated based on cell viability (n = 5). **d** Flow cytometric quantification of apoptotic cells, identified by Annexin V-FITC/PI staining, in H22-NR and H22-LR cells treated with lenvatinib (20 μM) for 48 h (n = 3). **e** Venn diagram of differential expressed genes between clinical lenvatinib-sensitive and lenvatinib-resistant HCC patient cohorts (HCC-NR and HCC-LR) and H22-LR/H22-NR murine tumor cohorts. **f** Expression of RPRD1A mRNA in H22-NR and H22-LR cells using RT-qPCR (n = 3). **g** Expression of RPRD1A protein in H22-LR/H22-NR tumors using western blot analysis. Data are presented as mean ± SEM, statistical significance: ns. not significant, *p < 0.05, and **p < 0.01

To elucidate the molecular basis of lenvatinib resistance, we established a syngeneic subcutaneous HCC mouse model with acquired therapeutic resistance via an intermittent high-dose lenvatinib administration regimen (Fig. 1a). Throughout the observation period, tumor volumes in the control group exhibited progressive growth. Mice receiving a single course of lenvatinib exhibited marked initial tumor regression, followed by rapid regrowth during the PBS maintenance phase. In contrast, the chronically lenvatinib-treated group displayed significant tumor suppression during treatment (days 10–20), with tumor growth arrested at a plateau. Notably, following two additional lenvatinib injections administered after day 20, this intensive rechallenge failed to effectively constrain tumor progression, confirming the establishment of a robust lenvatinib-resistant phenotype (Fig. 1b).

Based on this model, we subsequently isolated and cultured two sets of these tumor cells: a lenvatinib-sensitive cell line (H22-NR) derived from tumors in the control group and a lenvatinib-resistant cell line (H22-LR) derived from tumors chronically treated with lenvatinib. Drug sensitivity assays revealed a much higher IC50 for lenvatinib in H22-LR cells (18.99 μM) compared to H22-NR cells (6.50 μM), pharmacodynamically confirming lenvatinib resistance (Fig. 1c). Consistently, flow cytometry analysis revealed significantly reduced apoptosis in H22-LR cells following 48-h exposure to 20 μM lenvatinib, further validating the resistant phenotype (Fig. 1d).

We next performed RNA sequencing (RNA-seq) on tumor tissues from the control and lenvatinib-resistant groups (n = 3 per group). To identify conserved molecular signatures associated with lenvatinib resistance, we further integrated analysis of these datasets with transcriptome profiles from a clinical cohort of lenvatinib-treated HCC patients with documented resistance. Through this cross-cohort integration, six overlapping differentially expressed genes (TPPP3, PER1, SAA3, CRIP1, ENTPD3, RPRD1A) were identified (Fig. 1e). Consistently, RT-qPCR confirmed significant upregulation of RPRD1A in H22-LR cells relative to H22-NR cells. Furthermore, western blotting analysis demonstrated a 2.3-fold increase in RPRD1A protein levels in lenvatinib-resistant tumors compared to control tumors (Fig. 1f-g). Collectively, these findings suggest that RPRD1A plays a key role in mediating acquired resistance to lenvatinib in HCC.

### RPRD1A is involved in lenvatinib resistance in HCC

**Fig. 2:**
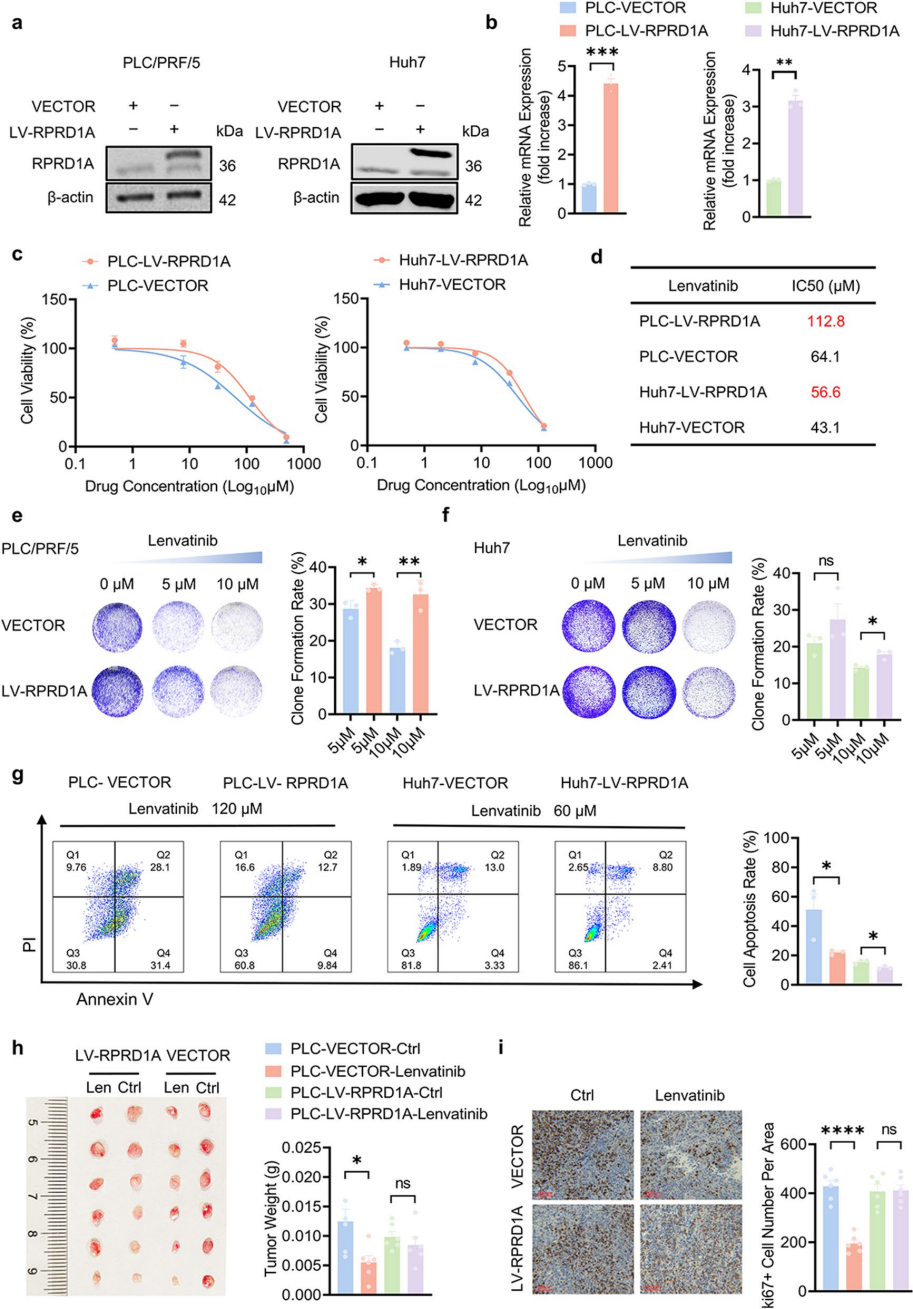
RPRD1A enhances lenvatinib resistance in HCC. **a** Expression of RPRD1A protein via western blot analysis.** b** Expression of RPRD1A mRNA in PLC-LV-RPRD1A, Huh7-LV-RPRD1A cells and their control cells (n = 3). **c** Dose–response curves of PLC-LV-RPRD1A (left panel) and Huh7-LV-RPRD1A cells (right panel) and their control cells treated with lenvatinib for 48 h (n = 5). **d** Lenvatinib IC50 values of (C) were calculated based on cell viability. **e–f** Representative images and statistical analysis of long-term colony formation assay of PLC-LV-RPRD1A (left panel), Huh7-LV-RPRD1A (right panel) and their control cells. Cells were grown in the absence or presence of lenvatinib at the indicated concentrations for 14 days (n = 3). **g** Flow cytometric quantification of apoptotic cells, identified by Annexin V-FITC/PI staining, in PLC-LV-RPRD1A (left panel), Huh7-LV-RPRD1A (right panel) and their control cells treated with lenvatinib at the indicated concentrations for 48 h (n = 3). **h** Syngeneic BALB/c mice were subcutaneously injected with PLC-LV-RPRD1A or PLC-VECTOR cells. Photographs of tumors and tumor weights of mice in the xenograft models are shown (n = 6). **i** Representative Ki67 IHC images in tumors of (**h**), along with statistical graphs of Ki67 IHC result analysis (n = 6). Scale bar, 200 μm. Data are presented as mean ± SEM, statistical significance: ns. not significant, *p < 0.05, **p < 0.01, ***p < 0.001, and ****p < 0.0001

To elucidate the functional role of RPRD1A in mediating lenvatinib resistance, we generated lentiviral-transduced RPRD1A-overexpressing cell lines alongside their respective vector controls (Fig. 2a-b, Fig. S1). Cell viability assays confirmed that RPRD1A-overexpressing cells exhibited significantly enhanced lenvatinib resistance compared to their control counterparts. Specifically, the half-maximal inhibitory concentration (IC50) of lenvatinib was increasing from 64.1 μM to 112.8 μM in PLC/PRF/5 cells, and from 43.1 μM to 56.6 μM in Huh7 cells (Fig. 2c-d). Consistently, upon exposure to lenvatinib, RPRD1A-overexpressing cells exhibited significantly enhanced clonogenic survival capacity and a significant reduction in apoptosis rates relative to the controls (Fig. 2e-g).

We next assessed whether high expression of RPRD1A can reduce the efficacy of lenvatinib in vivo. In mouse xenograft models, lenvatinib significantly suppressed tumor growth in PLC-VECTOR grafts but exerted no appreciable inhibitory effect on PLC-LV-RPRD1A grafts, indicating that RPRD1A overexpression induces acquired lenvatinib resistance (Fig. 2h). Immunohistochemical analysis revealed that lenvatinib markedly reduced Ki67⁺ proliferating cells in the PLC-VECTOR tumors but not in the PLC-LV-RPRD1A tumors (Fig. 2i). These in vivo findings confirm that RPRD1A overexpression directly drives lenvatinib resistance in HCC. For loss-of-function validation, we knocked down RPRD1A using two independent siRNAs (siRPRD1A#1 and siRPRD1A#2). Immunoblotting confirmed that both siRNAs achieved significant RPRD1A protein depletion, relative to scramble siRNA controls (Fig. S2a). RPRD1A knockdown significantly reduced the lenvatinib IC50 (Fig. S2b-c), inhibited clonogenicity (Fig. S2d-e), and increased the apoptotic rate compared to scramble siRNA controls (Fig. S2f). These results substantiate that RPRD1A silencing enhances lenvatinib sensitivity in HCC cells.

### RPRD1A transcriptionally regulates ITGA5 expression to drive resistance in HCC

**Fig. 3:**
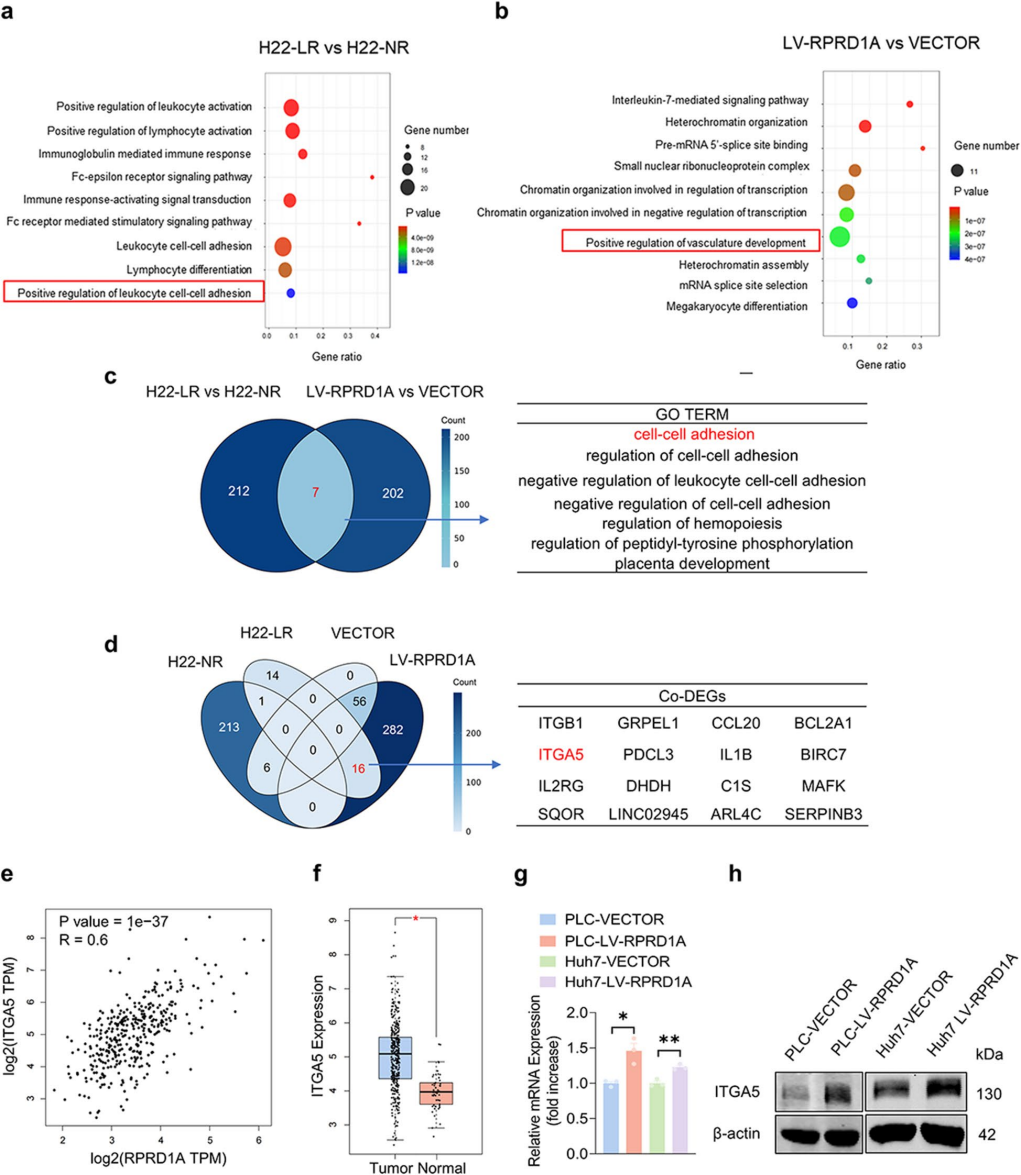
RPRD1A promotes ITGA5 expression in HCC. **a** Top GO terms of the differentially expressed genes of the tumor cells between H22-LR and H22-NR mice based on RNA-seq data.** b** Top GO terms of the differentially expressed genes of PLC-LV-RPRD1A and PLC-VECTOR cells based on RNA-seq data. **c** Integrated analysis of data from (**a**) and (**b**), highlighting the 7 commonly upregulated signaling pathways identified. **d** Integrated analysis of data from (**a**) and (**b**) reveals 16 commonly upregulated genes. **e** Correlation analysis of RPRD1A and ITGA5 expression levels in HCC based on data from the GEPIA2.0 database. **f** ITGA5 mRNA levels in HCC tissues versus adjacent normal liver tissues based on data from the GEPIA2.0 database. **g** Expression of ITGA5 mRNA in PLC-LV-RPRD1A, Huh7-LV-RPRD1A and their control cells via RT-PCR (n = 3). **h** Expression of ITGA5 protein in PLC-LV-RPRD1A, Huh7-LV-RPRD1A and their control cells via western blot analysis. Data are presented as mean ± SEM, statistical significance: ns. not significant, *p < 0.05, and **p < 0.01

We next investigated whether RPRD1A contributes to drug resistance by activating the canonical target-related signaling pathways of lenvatinib (e.g., PI3K-AKT-mTOR and MEK-ERK pathways). However, our results demonstrated that neither pathway exhibited significant activation following RPRD1A overexpression, ruling out these classical axes as downstream mediators (Fig. S3). To further delineate alternative downstream resistance mechanisms, we performed transcriptomic profiling of tumor tissues from lenvatinib-resistant (H22-LR) and lenvatinib-sensitive (H22-NR) mice. Gene Ontology (GO) enrichment analysis revealed that cell adhesion-related pathways were significantly upregulated in the resistant tumors (Fig. 3a). Consistently, RPRD1A-overexpressing cells showed enrichment of vascular developmental pathways (Fig. 3b). Integrated analysis of these two datasets aforementioned (resistant vs. sensitive tumors, and RPRD1A-overexpressing vs. control cells) identified seven conserved biological processes: cell–cell adhesion, regulation of cell–cell adhesion, hemopoiesis regulation, peptidyl-tyrosine phosphorylation regulation, and placenta development (Fig. 3c). Additionally, 16 shared differentially expressed genes were identified, including *ITGA5, ITGB1, GRPEL1, CCL20, BCL2A1, PDCL3, IL1B, BIRC7, IL2RG, DHDH, C1S, MAFK, SQOR, LINC02945, ARL4C,* and *SERPINB3* (Fig. 3d). Notably, *ITGA5* emerged as a central node functionally linking cell adhesion and vascular development pathways, suggesting its functional centrality within RPRD1A-mediated resistance.

Furthermore, analysis of The Cancer Genome Atlas (TCGA) HCC cohort revealed a strong positive correlation between RPRD1A and ITGA5 expression (Pearson correlation coefficient r = 0.6, *p* < 0.001; Fig. 3e). Clinically, ITGA5 expression was significantly elevated in HCC tissues compared to normal liver tissues (*p* < 0.05; Fig. 3f). Mechanistic validation confirmed that RPRD1A overexpression significantly upregulated ITGA5 expression at both the mRNA and protein levels in HCC cells (Fig. 3g-h). Collectively, these findings strongly support that the RPRD1A/ITGA5 axis plays a critical regulatory role in lenvatinib resistance.

### RPRD1A transcriptionally activates ITGA5 through competitive binding with RNA Pol II and c-JUN-dependent mechanisms

**Fig. 4:**
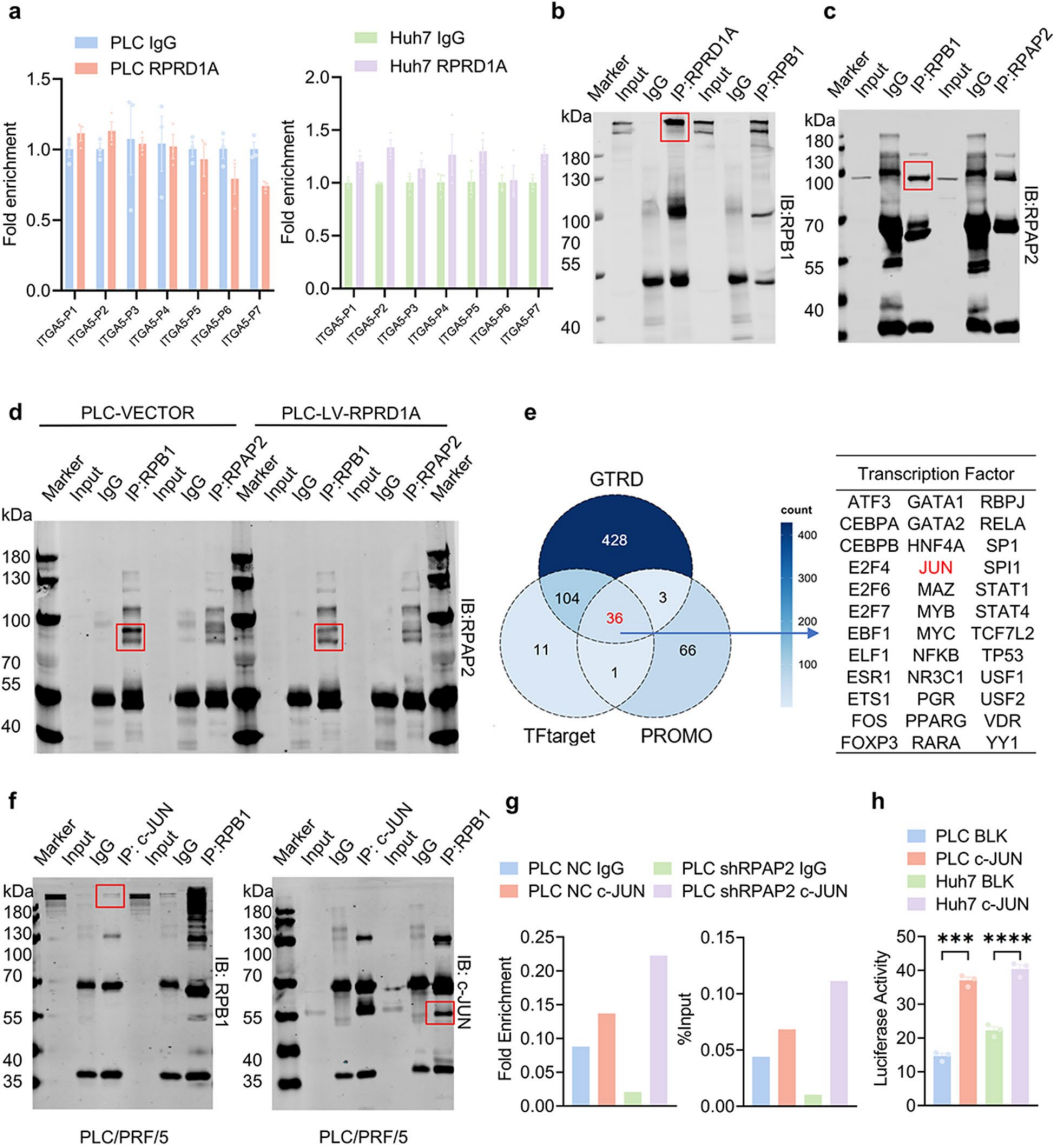
RRPD1A positively regulates ITGA5 expression by competitively binding RNA pol II with RPAP2 leading to lenvatinib resistance. **a** ChIP assays showing that RPRD1A cannot directly bind to the promoter region of ITGA5 in PLC/PRF/5 and Huh7 cells (n = 3). **b** Co-IP analysis showing that the RPRD1A protein could bind to RPB1, the largest subunit of RNA pol II in PLC/PRF/5 cells.** c** Co-IP analysis showing that the RPAP2 protein could bind to RPB1 in PLC/PRF/5 cells. **d** Co-IP analysis showing attenuated interaction of RPAP2 with RPB1 in PLC-LV-RPRD1A compared to PLC-VECTOR. **e** Bioinformatic screening using GTRD, PROMO, and TFtarget platforms identified 36 candidate ITGA5 transcription factors. **f** Co-IP analysis showing that the c-JUN protein can bind to RPB1 in PLC/PRF/5 cells. **g** ChIP assays demonstrating enhanced c-JUN-mediated ITGA5 transcriptional activation following RPAP2 knockdown (n = 3). **h** Dual-luciferase reporter analysis showing significantly increased ITGA5 transcriptional activity upon c-JUN overexpression (n = 3). Data are presented as mean ± SEM, statistical significance: ns. not significant, ***p < 0.001, ****p < 0.0001

Given that RPRD1A participates in transcription elongation via interactions with RNA polymerase II (RNA Pol II) [17], we explored its potential role in ITGA5 expression. Chromatin immunoprecipitation (ChIP) assays revealed no significant enrichment of RPRD1A at the canonical *ITGA5* promoter region, ruling out direct transcriptional activation by RPRD1A (Fig. 4a).

Consistent with prior evidence that RPAP2 binds RNA Pol II to mediate transcriptional repression [18], we hypothesized that RPRD1A may competitively interact with RPAP2 for binding to RNA Pol II. Co-immunoprecipitation (Co-IP) confirmed that both RPRD1A and RPAP2 directly interact with RPB1, the largest subunit of RNA Pol II (Fig. 4b-c, and Fig. S4a-b). Crucially, competitive binding assays demonstrated that RPRD1A overexpression significantly reduced RPAP2-RPB1 complex formation by outcompeting RPAP2 for RPB1 binding. (Fig. 4d and Fig. S4c). To identify transcription factors (TFs) that might act downstream of this RPRD1A-RPAP2-RNA Pol II axis to regulate *ITGA5*, we performed bioinformatic screening using GTRD, PROMO, and TFtarget databases, which predicted 36 candidate transcription factors potentially regulating *ITGA5* transcription. Based on the following three criteria, we further narrowed down the candidate transcription factors potentially responsible for RPRD1A-mediated ITGA5 upregulation: (1) the factor must be known to play critical functional roles in HCC; (2) it should be associated with cell adhesion processes or the integrin signaling pathway; and (3) direct regulation of ITGA5 by this factor must have been previously documented in the literature. Applying these criteria, c-JUN—a core component of the AP-1 transcription factor complex—emerged as the most compelling candidate. Its well-established oncogenic functions in HCC, coupled with its reported involvement in integrin-mediated signaling and its ability to directly bind the ITGA5 promoter, strongly supported its selection for subsequent experimental validation [19] (Fig. 4e). Mechanistic validation confirmed that c-JUN binds RPB1 (Fig. 4f and Fig. S4d) and the ITGA5 promoter (Fig. 4g), and further enhances ITGA5 promoter activity (Fig. 4g-h and Fig. S4e). Notably, RPAP2 knockdown significantly augmented c-JUN-mediated ITGA5 transcription (Fig. 4h and Fig. S4e-f). In brief, these results indicate that RPRD1A competitively displaces RPAP2 from RNA Pol II, thereby alleviating transcriptional repression and facilitating c-JUN-mediated activation of ITGA5 expression.

### The RPRD1A/ITGA5 axis induces resistance via the FAK pathway

**Fig. 5:**
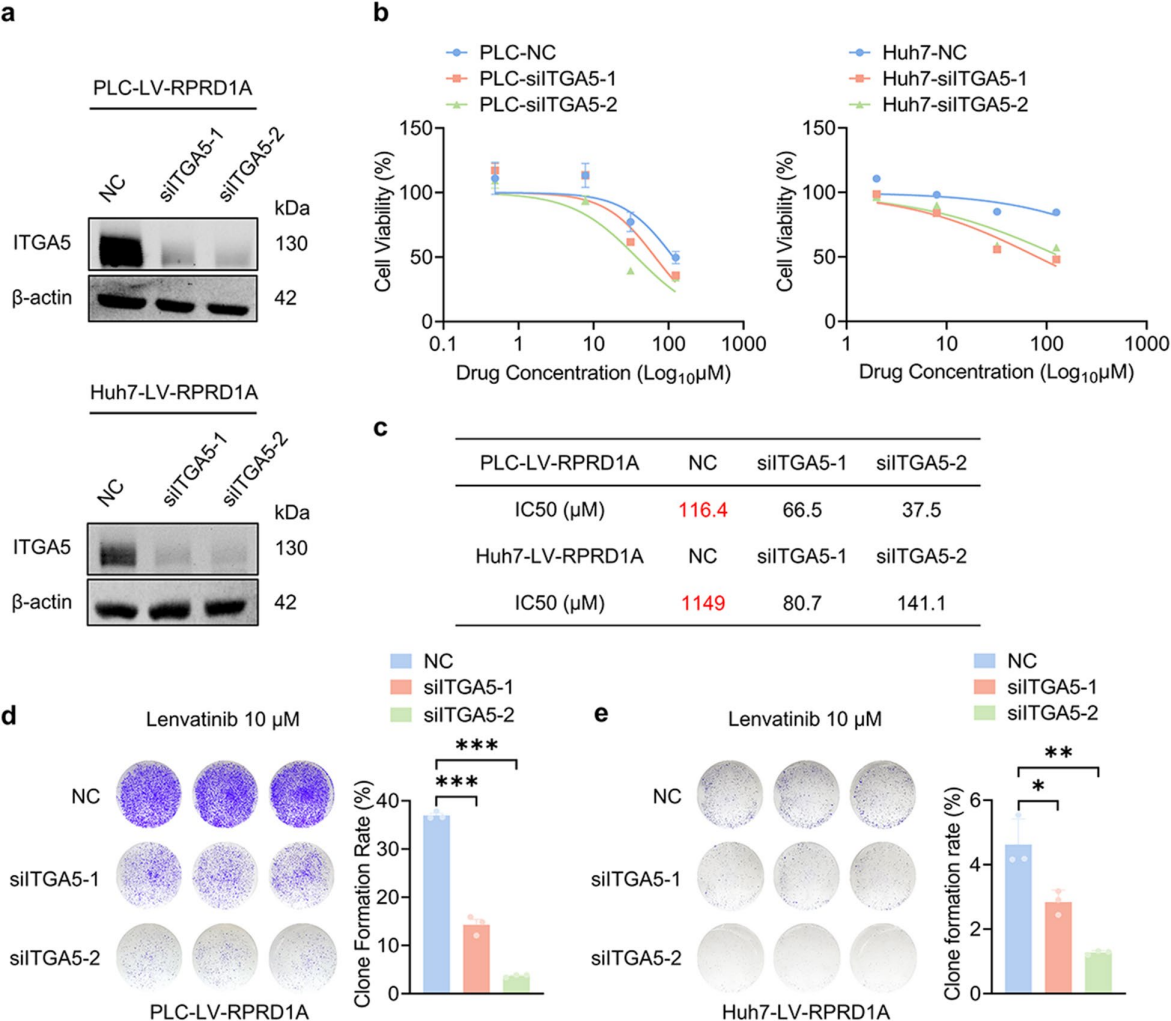
Targeting the ITGA5 resensitizes lenvatinib in HCC. **a** Expression of ITGA5 protein in PLC-LV-RPRD1A (upper panel) and Huh7-LV-RPRD1A (lower panel) cells transfected with siITGA5 via western blot analysis.** b** Dose–response curves of siITGA5-transfected PLC-LV-RPRD1A (left panel) and Huh7-LV-RPRD1A (right panel) cells and their control cells treated with lenvatinib for 48 h (n = 3). **c** lenvatinib IC50 values of (**b**) were calculated based on cell viability. **d-e** Representative images and statistical analysis of long-term colony formation assay of ITGA5-knockdown PLC-LV-RPRD1A (left panel), Huh7-LV-RPRD1A cells (right panel) and their control cells. Cells were grown in the presence of lenvatinib at the indicated concentrations for 14 days (n = 3). Data are presented as mean ± SEM, statistical significance: ns. not significant, *p < 0.05, **p < 0.01, and ***p < 0.001

To delineate the functional role of ITGA5 in RPRD1A-mediated lenvatinib resistance, we knocked down ITGA5 in RPRD1A-overexpressing PLC/PRF/5 and Huh7 cells using two independent siRNAs. Efficient depletion of ITGA5 protein was verified by immunoblot analysis (Fig. 5a). Functional assessment demonstrated that ITGA5 silencing significantly resensitized RPRD1A-overexpressing cells to lenvatinib, as evidenced by reduced IC50 values relative to non-targeting siRNA-transfected controls (Fig. 5b-c). Consistent with these findings, colony formation assays revealed markedly impaired clonogenic capacity in ITGA5 knockdown cells upon lenvatinib treatment (Fig. 5d-e). These data indicate that targeting ITGA5 reverses RPRD1A-driven lenvatinib resistance in HCC.

**Fig. 6:**
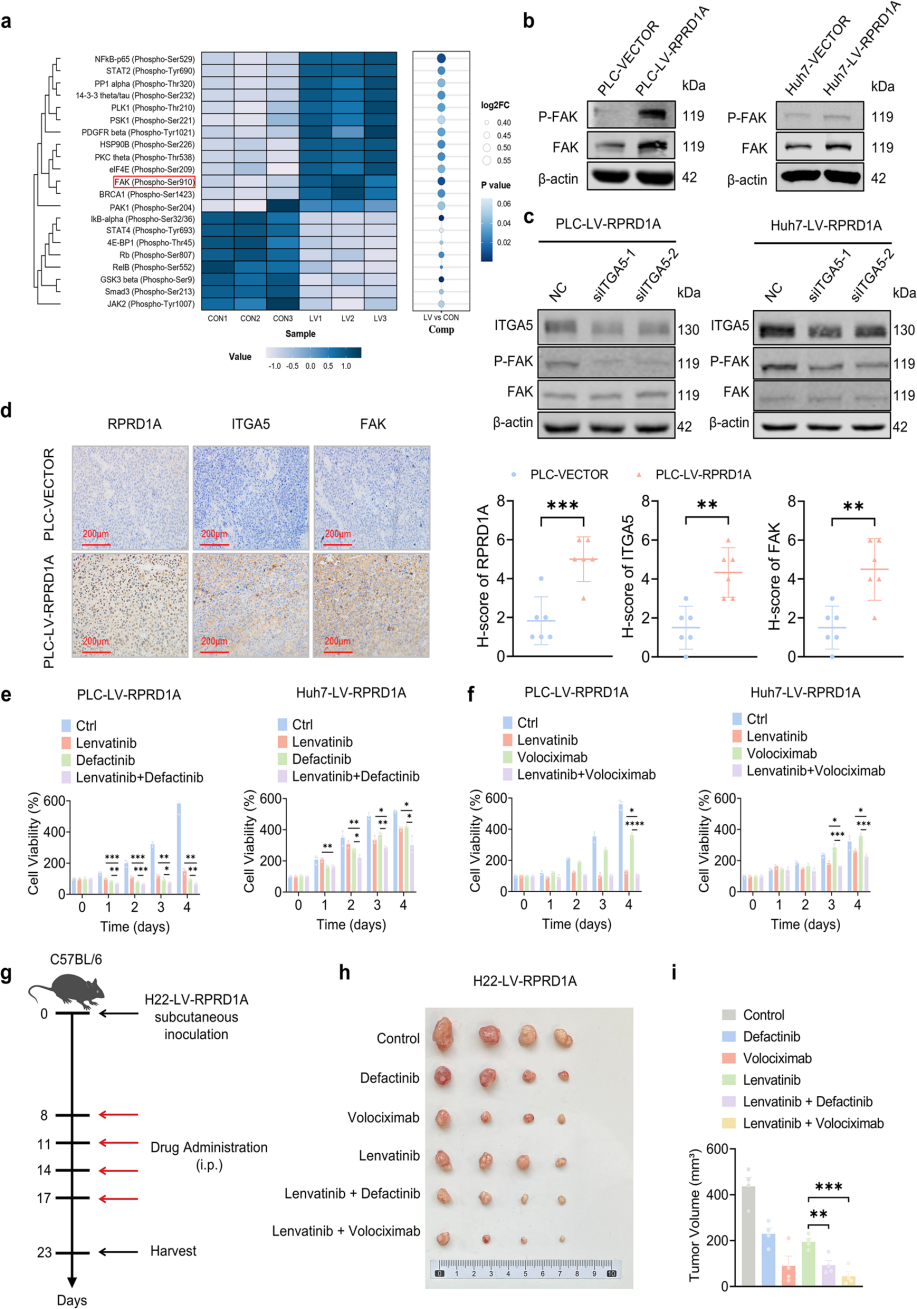
ITGA5 induces resistance to lenvatinib in RPRD1A-overexpressing HCC cells through the FAK pathway. **a** Heatmap showing phosphorylated protein differential expression between PLC-LV-RPRD1A and PLC-VECTOR cells. **b** Expression of phosphorylated FAK protein in PLC-LV-RPRD1A (left panel) and Huh7-LV-RPRD1A (right panel) cells via western blot analysis.** c** Expression of phosphorylated FAK protein in siITGA5-transfected PLC-LV-RPRD1A (left panel), Huh7-LV-RPRD1A (right panel), and their control cells via western blot analysis. **d** Representative images and statistical analysis of IHC staining of RPRD1A, ITGA5 and FAK in PLC-LV-RPRD1A and PLC-VECTOR mice tumors (n = 6), scale bar: 200 μm. **e** Time-course survival rates of PLC-LV-RPRD1A (left panel) and Huh7-LV-RPRD1A (right panel) cells treated with lenvatinib (30 μM), Donafenib (5 μM), their single-agent controls or the combination therapy, assessed at 24-h intervals over a 4-day period (n = 3). **f** Time-course survival rates of PLC-LV-RPRD1A (left panel) and Huh7-LV-RPRD1A (right panel) cells treated with lenvatinib (30 μM), Volociximab (100 nM), their single-agent controls or the combination therapy, assessed at 24-h intervals over a 4-day period (n = 3). **g** Experimental design of the treatment procedure. **h** Photographs of tumors of mice in the xenograft models (C57BL/6 mice bearing H22-LV-RPRD1A subcutaneous xenografts after treatment with PBS, lenvatinib (30 mg/kg), Donafenib (50 mg/kg), Volociximab (10 mg/kg), or their combinations) are shown (n = 4). **i** Tumor volumes of mice in the xenograft models in (**h**) are shown (n = 4). Data are presented as mean ± SEM, statistical significance: ns. not significant, *p < 0.05, **p < 0.01, ***p < 0.001, and ****p < 0.0001

Building upon the established RPRD1A/ITGA5 axis, we performed phosphoproteomic profiling to dissect the underlying downstream signaling mechanisms. Comparative analysis via CSP100 Plus protein array between PLC-LV-RPRD1A and PLC-VECTOR cells revealed significant enrichment of components within the FAK pathway, and phosphorylation quantification confirmed elevated levels of pY397-FAK (Fig. 6a, p < 0.05). Protein–protein interaction (PPI) network analysis constructed using the STRING database highlighted ITGA5 as a key regulator in the FAK signaling pathway (Fig. S5a). Given the well-documented role of ITGA5 in FAK activation via fibronectin binding [20], we hypothesized that ITGA5 mediates this pathway. Western blot analysis validated increased levels of phospho-FAK (Y397) in PLC-LV-RPRD1A and Huh7-LV-RPRD1A cells (Fig. 6b), while subsequent ITGA5 knockdown substantially reversed this FAK hyperphosphorylation phenotype (Fig. 6c). Importantly, these cellular findings were supported by clinical data: TCGA cohort analysis demonstrated a significant positive co-expression correlation between RPRD1A and FAK (r = 0.35, *p* < 0.001, Fig. S5b), as well as between ITGA5 and FAK (r = 0.21, *p* < 0.0001, Fig. S5c) in human HCC specimens. Consistent with these observations, immunohistochemical staining of xenograft tumors showed concomitant upregulation of both ITGA5 and FAK in PLC-LV-RPRD1A tumors relative to vector controls (Fig. 6d).

To assess the translational potential of targeting this signaling axis, we first evaluated the therapeutic efficacy of combination regimens in vitro. Co-treatment with lenvatinib and defactinib (a FAK inhibitor) significantly reduced cell viability compared to monotherapy (*p* < 0.05, Fig. 6e), paralleling the synergistic effect observed with lenvatinib plus volociximab (an ITGA5 inhibitor, *p* < 0.05, Fig. 6f). Critically, consistent with these in vitro findings, in vivo xenograft studies demonstrated that both combination therapies (lenvatinib + defactinib and lenvatinib + volociximab) achieved significantly greater tumor burden reduction than lenvatinib monotherapy (Fig. 6g-i).

**Fig. 7:**
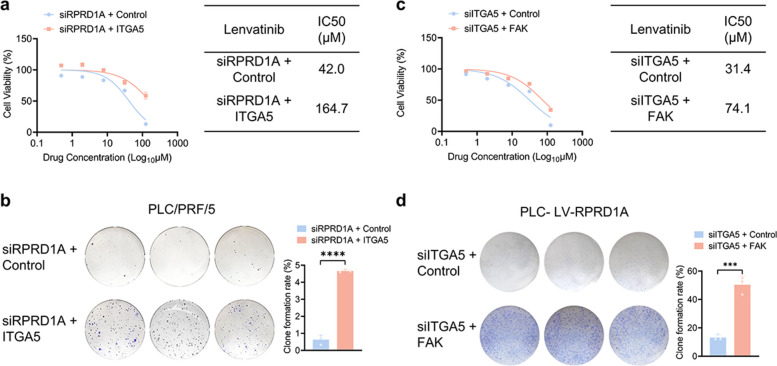
Rescue experiments confirm the RPRD1A–ITGA5–FAK axis in mediating lenvatinib resistance. **a** Dose–response curves and IC50 values of lenvatinib in PLC/PRF/5 cells co-transfected with RPRD1A siRNA and ITGA5 overexpression plasmid or control constructs, following 48 h treatment (n = 3). **b** Representative images and statistical analysis of long-term colony formation assay of RPRD1A-knockdown and ITGA5 overexpression PLC-LV-RPRD1A and their control cells. Cells were grown in the presence of lenvatinib at the indicated concentrations for 14 days (n = 3). **c** Dose–response curves and IC50 values of lenvatinib in PLC‑LV‑RPRD1A cells co-transfected with ITGA5 siRNA and CA-FAK or control FAK plasmid, following 48 h treatment (n = 3). **d** Representative images and statistical analysis of long-term colony formation assay of ITGA5-knockdown and FAK overexpression PLC-LV-RPRD1A and their control cells. Cells were grown in the presence of lenvatinib at the indicated concentrations for 14 days (n = 3). Data are presented as mean ± SEM, statistical significance: ns. not significant, ***p < 0.001, and ****p < 0.0001

To further confirm that ITGA5 and FAK serve as key downstream effectors of RPRD1A, we reconstituted ITGA5 expression in RPRD1A-knockdown cells and re-expressed FAK in ITGA5-knockdown cells. The results demonstrated that ITGA5 overexpression partially reversed lenvatinib hypersensitivity induced by RPRD1A depletion. Similarly, FAK overexpression restored lenvatinib resistance that had been compromised by ITGA5 knockdown (Fig. 7 and Fig. S6). These data functionally validate the RPRD1A–ITGA5–FAK downstream regulatory cascade.

Altogether, these data establish a coherent signaling hierarchy in which RPRD1A drives ITGA5-dependent FAK hyperactivation, ultimately governing the therapeutic response to lenvatinib in HCC cells and xenograft models.

### Clinical correlation between RPRD1A/ITGA5 expression and lenvatinib therapeutic response

**Fig. 8:**
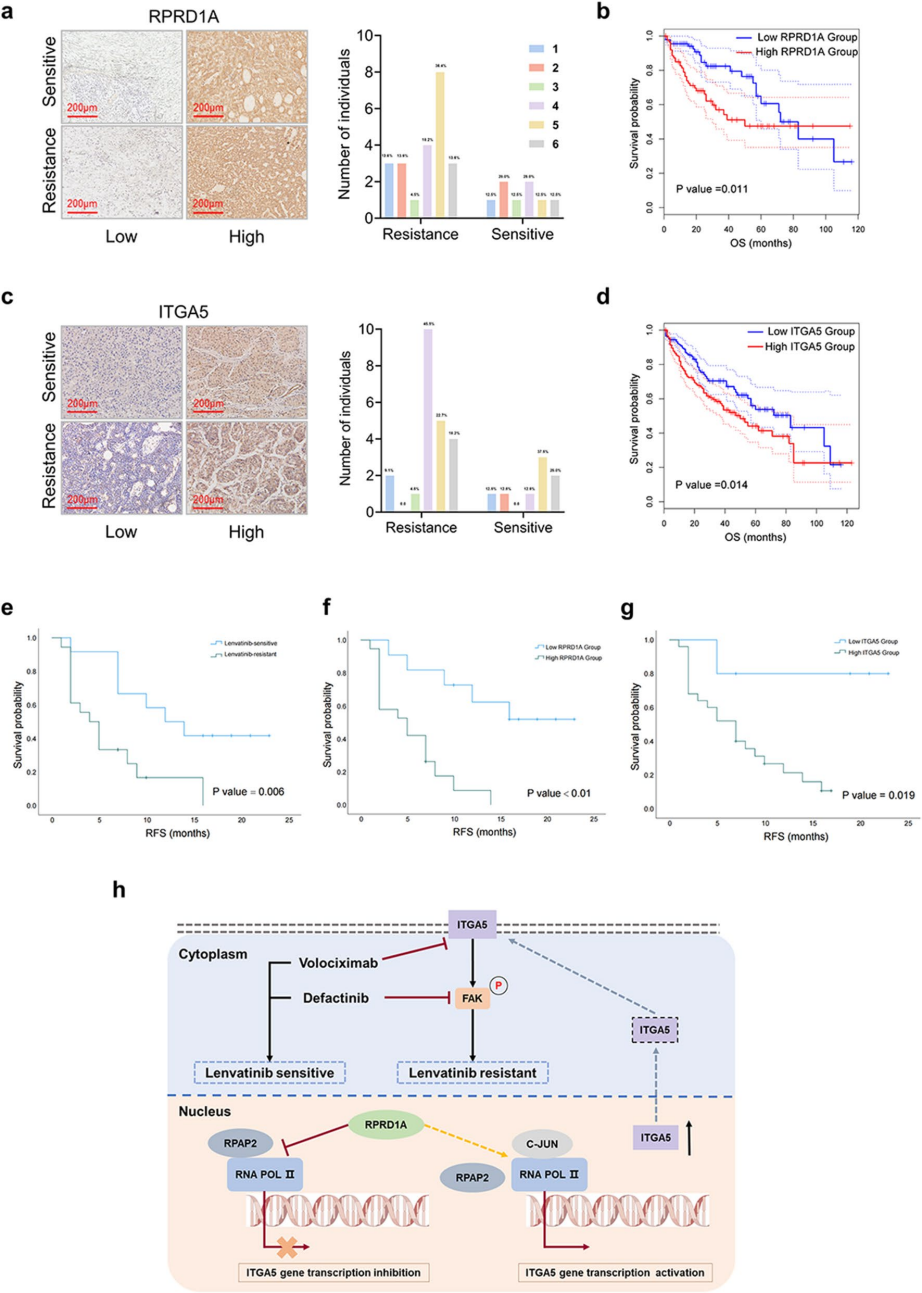
RPRD1A and ITGA5 expression levels correlate with lenvatinib clinical responsiveness. **a** Representative images and statistical analysis of IHC staining of RPRD1A expression in HCC tissues from clinically lenvatinib-sensitive and lenvatinib-resistant patients, scale bar, 200 μm. **b** Survival analysis of RPRD1A expression levels and prognosis in HCC based on data from the GEPIA2.0 database. **c** Representative images and statistical analysis of IHC staining of ITGA5 expression in HCC tissues from clinically lenvatinib-sensitive and lenvatinib-resistant patients, scale bar, 200 μm. **d** Survival analysis of ITGA5 expression levels and prognosis in HCC based on data from the GEPIA2.0 database. **e** Kaplan–Meier analysis of lenvatinib sensitivity and RFS in HCC patients. **f** Kaplan–Meier analysis of RPRD1A expression and RFS in HCC patients. **g** Kaplan–Meier analysis of ITGA5 expression and RFS in HCC patients. **h** Schematic overview of the present study: RPRD1A positively regulates ITGA5 expression by competitively binding to RNA Pol II and interfering with RPAP2, leading to activation of the focal adhesion kinase (FAK) signaling pathway and subsequent non-canonical induction of lenvatinib resistance

To evaluate the clinical relevance of the RPRD1A-ITGA5-FAK axis in lenvatinib resistance, we analyzed clinical samples from HCC patients. Specifically, the expression levels of RPRD1A and ITGA5 were examined in HCC tumors obtained from 30 patients who received lenvatinib. Although RPRD1A expression was heterogeneous in both lenvatinib-sensitive and -resistant subgroups, the resistant subgroup exhibited a significantly higher proportion of tumors with high RPRD1A expression (63.3% vs. 36.7%, Fig. 8a). Consistently, the objective response rate (ORR) was markedly lower in RPRD1A-high patients (21.1% [4/19]) compared to RPRD1A-low patients (36.3% [4/11]; Table S1). In addition, high RPRD1A expression was associated with poor prognosis in HCC patients (*p* < 0.05; Fig. 8b). Similarly, the resistant subgroup showed a substantially higher frequency of tumors with high ITGA5 expression (83.3% vs. 16.7%, Fig. 8c), which corresponded to a reduced clinical response and worse prognosis (ITGA5-high ORR: 24.0% [6/25] vs. 40.0% [2/5] in ITGA5-low; Table S1, Fig. 8d). Further Kaplan–Meier analysis demonstrated that patients with a poor response to lenvatinib (Fig. 8e, Log-rank *p* = 0.006), high RPRD1A expression (Fig. 8f, Log-rank *p* < 0.01), or high ITGA5 expression (Fig. 8g, Log-rank *p* = 0.019) had a significantly shorter PFS. To determine whether these factors were independently prognostic, a multivariate Cox proportional hazards model was applied. After adjustment for age, sex, and tumor burden, both high RPRD1A expression (HR = 4.886, 95% CI: 1.538–15.521, *p* = 0.007) and high ITGA5 expression (HR = 4.993, 95% CI: 1.647–38.542, *p* = 0.023) emerged as significant independent predictors of poor PFS (Table S2).

Taken together, these clinical observations confirm RPRD1A and ITGA5 as potential biomarkers capable of stratifying HCC patients by predicting their response to lenvatinib, thereby bridging our experimental mechanistic insights to the clinical setting of lenvatinib resistance (Fig. 8h).

## Discussion

As a cornerstone of systemic therapy for advanced HCC, lenvatinib has emerged as the most widely used first-line targeted therapeutic agent in clinical practice. However, the prevalence of tumor resistance significantly undermines its antitumor efficacy, frequently resulting in disease progression. Currently, only a limited number of molecules have been implicated in lenvatinib resistance, including epidermal growth factor receptor (EGFR) [21, 22], YTH domain family protein 1 (YTHDF1) [23], histone deacetylase (HDAC) [24], frizzled class receptor 10 (FZD10) [25], lysosomal-associated membrane protein transmembrane 5 (LAPTM5) [26], and cyclin-dependent kinase 6 (CDK6) [27]. Using a mouse model of acquired lenvatinib resistance, we identified RPRD1A as a key mediator of this resistance and unraveled a novel mechanism by which RPRD1A promotes lenvatinib resistance through the “RPRD1A-ITGA5-FAK” signaling axis. These findings not only validate RPRD1A and ITGA5 as potential biomarkers for predicting lenvatinib response but also lay a conceptual foundation for developing novel combination therapies to overcome lenvatinib resistance.

RPRD1A, alias P15RS, first cloned from human melanoma cell line A375 [28], is a member of the RPRD family (including RPRD1A, RPRD1B, RPRD2) that regulates cell cycle progression and transcriptional activity. Located on chromosome 18q12, it acts via two key mechanisms: interacting with CDK4 inhibitor B (CDKN2B) to negatively regulate G1/S phase transition, and forming homodimers or heterodimers to modify RNA Pol II’s CTD by binding to RPB1, thus modulating transcription [29]. Studies have shown that RPRD1A is downregulated in breast cancer, inversely correlating with advanced N/M stage, tumor recurrence and poor survival [30]; it exerts tumor-suppressive effects by inhibiting the Wnt/β-catenin pathway and cell cycle-related genes [31]. Notably, its function is context-dependent: while overexpression inhibits melanoma cell clonogenicity and migration [32], it promotes HCC progression. Our recent study found RPRD1A is upregulated in HCC tissues and portal vein tumor thrombi, enhancing antioxidant responses and platinum resistance via the “RPRD1A-TRIM21-p62” axis, which correlates with poor prognosis [33]. However, its role in lenvatinib resistance is unclear. In this study, we first identified a novel NRF2-independent RPRD1A-ITGA5-FAK axis mediating lenvatinib resistance. To clarify the association between this axis and the NRF2 pathway, we detected key NRF2 pathway proteins (NQO1, HO-1) in RPRD1A-overexpressing HCC cells; lenvatinib treatment did not significantly alter their expression (Fig. S7a–b). Similarly, siRNA-mediated knockdown of TRIM21 or p62 did not affect lenvatinib resistance (Fig. S7c-d). These results indicate that RPRD1A drives lenvatinib resistance mainly through the ITGA5-FAK axis, which is mechanistically independent of or parallel to the NRF2 pathway. This suggests RPRD1A may serve as a molecular hub, selectively activating distinct downstream signaling modules in response to different therapeutic stresses (e.g., chemotherapy, anti-angiogenic TKI treatment) to promote tumor cell survival.

Here, we unraveled that RPRD1A upregulates ITGA5 expression through c-JUN-dependent transcriptional activation, in which RPRD1A competitively displaces RPAP2 from RNA Pol II, confirming ITGA5 as a key downstream target of RPRD1A. As a core integrin family member, ITGA5 forms the α5β1 heterodimer with ITGB1-encoded β1 subunit, regulating cell adhesion, migration and signal transduction by recognizing arginine-glycine-aspartate (RGD) motifs in extracellular ligands, thereby mediating intercellular communication and extracellular matrix (ECM) remodeling [34–36]. As a fibronectin/fibrinogen receptor, integrin α5β1 promotes tumor angiogenesis, invasion and metastasis via FAK/PI3K/AKT signaling, correlating with poor prognosis across multiple malignancies [37, 38]. Accumulating evidence confirms ITGA5’s oncogenic role: its upregulation enhances proliferation, metastasis, invasion or EMT inbreast cancer [39], enhances invasion and epithelial–mesenchymal transition (EMT) in colorectal cancer [19, 40, 41], facilitates peritoneal dissemination in ovarian cancer [42]; boosts proliferation and migration in non-small cell lung cancer (NSCLC) [42, 43], and correlates with worse survival in gastric cancer (GC) [44]. In HCC, the ITGA5/ITGB1 complex induces hypoxia and vasculogenic mimicry, contributing to sorafenib resistance [45]. In the present study, we confirmed that high ITGA5 expression impairs lenvatinib efficacy in HCC, while siRNA-mediated ITGA5 knockdown substantially restores lenvatinib sensitivity. Moreover, combining the ITGA5 inhibitor volociximab with lenvatinib synergistically reduced HCC tumor burden, identifying ITGA5 as a critical regulator of lenvatinib resistance and a promising combinatorial therapy candidate.

Using protein chip technology, we found that elevated RPRD1A and ITGA5 expression effectively activates downstream FAK signaling. As a non-receptor tyrosine kinase, FAK integrates intra- and extracellular signals to regulate key cellular processes (adhesion, migration, survival) and plays a vital role in tumor microenvironment (TME) remodeling [46]. Upon ECM binding to integrins (e.g., α5β1), FAK is recruited to focal adhesions, where dimerization relieves autoinhibition, followed by autophosphorylation at Y397 and subsequent SRC-mediated phosphorylation at Y576/Y577; this dual phosphorylation triggers an activation loop that promotes proliferation, migration and survival [47]. Aberrant FAK hyperactivation is frequently observed in HCC and linked to multi-drug resistance [48]. Preclinical studies have shown that FAK inhibitors can reverse chemotherapy resistance in lung cancer [49, 50], targeted therapy resistance in breast cancer [51], temozolomide resistance in glioblastoma [52], and paclitaxel resistance in ovarian cancer [53]. Notably, FAK signaling mediates sorafenib resistance in HCC by maintaining tumor cell adhesion and vasculogenic mimicry [54]. Consistent with a previous study that FAK overexpression induces lenvatinib resistance [55], our results showed combining the FAK inhibitor defactinib with lenvatinib significantly enhanced lenvatinib sensitivity in low-responsive HCC cells, which was validated in vivo (marked tumor volume reduction vs lenvatinib monotherapy). Additionally, FAK modulates the TME and immune evasion, affecting anti-tumor T cell responses [56]. A recent study demonstrated that FAK inhibition enhances HCC sensitivity to anti-PD-1 therapy by suppressing angiogenesis/fibrosis and promoting CD8 + T cell infiltration [57]. Thus, FAK inhibition potentiates lenvatinib efficacy and sensitizes HCC to immunotherapy, making FAK inhibition combined with targeted/immunotherapy a novel, clinically feasible strategy for HCC.

Besides, we explored the correlation between RPRD1A/ITGA5 expression levels and clinical response to lenvatinib monotherapy. In a well-defined cohort of HCC patients receiving single-agent lenvatinib, the resistant group exhibited a significantly higher proportion of patients with high RPRD1A expression compared to the sensitive group. Similarly, high ITGA5 expression was markedly more prevalent in the resistant cohort than in the sensitive cohort. Remarkably, objective response rate (ORR) was significantly lower in patients with high expression of either RPRD1A (21.1% vs. 36.3%) or ITGA5 (24.0% vs. 40.0%) relative to those with low expression. These critical clinical findings mark the first real-world validation of a direct association between high RPRD1A/ITGA5 expression and both intrinsic lenvatinib resistance and reduced therapeutic efficacy, supporting their potential as predictive biomarkers for lenvatinib response. This discovery furnishes critical evidence for prospectively identifying patients at high risk of lenvatinib resistance and optimizing combination therapy strategies.

Although our immunodeficient nude mouse model confirmed the RPRD1A–ITGA5–FAK axis’s role in lenvatinib resistance, it fails to assess its impact on the tumor immune microenvironment (TIME). Additionally, the function of RPRD1A has not been systematically validated in multiple lenvatinib-resistant cell lines, requiring further in vitro studies. Given lenvatinib’s clinical combination with immunotherapy, future work in immunocompetent mouse models is warranted to explore whether this axis drives resistance via remodeling an immunosuppressive TIME. Moreover, our intermittent high-dose in vivo model only recapitulates short-term resistance, differing from clinical resistance induced by sustained low-dose pressure. Long-term low-dose cell models or patient-derived organoids (PDO) will better simulate clinically acquired resistance.

In summary, our study demonstrates that RPRD1A/ITGA5 mediates lenvatinib resistance in HCC by activating the FAK signaling pathway. Mechanistically, RPRD1A competitively displaces RPAP2 from RNA Pol II, thereby relieving transcriptional repression and enabling c-JUN-mediated activation of ITGA5 transcription. Subsequent upregulation of ITGA5 triggers activation of the FAK signaling axis, ultimately reinforcing lenvatinib resistance. Targeting the RPRD1A-ITGA5-FAK axis represents a promising therapeutic approach to substantially improve the clinical efficacy of lenvatinib in advanced HCC.

## Materials and methods

### Clinical samples

Thirty patients with advanced HCC were recruited from our institution, all staged as BCLC class C without prior systemic therapy (including sorafenib or other targeted/immunotherapeutic agents). Definitions of lenvatinib sensitivity/resistance and detailed clinicopathological data are summarized in Supplementary Tables S3–S5.

Inclusion criteria: pathologically confirmed HCC, first-line lenvatinib therapy (monotherapy or combination with immunotherapy), and complete clinical/follow-up data. Exclusion criteria: concurrent active malignancies, prior systemic treatment, uncontrolled comorbidities, insufficient treatment duration, or incomplete clinical data. The study was approved by the hospital ethics committee, with written informed consent obtained from all participants.

### Extraction of primary tumor cells

Primary tumor cells were isolated from the murine tumor tissues using mechanical and enzymatic dissociation. Briefly, excised tumors were minced and digested in collagenase D solution for 1–2 h at 37 °C. The resulting suspension was sequentially filtered through a 100 μm cell strainer, treated with red blood cell lysis buffer for erythrocyte removal, and centrifuged at 900 × rpm for 3 min. Cells were resuspended in complete medium (RPMI-1640 (#L210KJ, BasalMedia) supplemented with 20% FBS (#C04001-500, VivaCell), 1 μM dexamethasone (#D-085, Sigma), 5 μg/mL insulin (#PHR8925, Sigma), and 1% antibiotic–antimycotic (#S120JV, BasalMedia) and cultured at 37 °C under 5% CO₂.

### Cell lines and culture

H22 (#CL-0341), Huh7 (#CL-0120), and PLC/PRF/5 (#CL-0415) cell lines were purchased from Wuhan Pricella Biotechnology with STR verification and cultured at 37 °C in 5% CO₂ with medium changes every other day. Parental H22 cells were maintained in RPMI-1640 containing 10% FBS and 1% antibiotics, while H22-LR and H22-NR were cultured in RPMI-1640 supplemented with 20% FBS, 1 μM dexamethasone, 5 μg/mL insulin, and 1% antibiotics. Huh7, PLC/PRF/5, and their transduced derivatives were grown in DMEM (#L110KJ, BasalMedia) with 10% FBS and 1% antibiotics.

H22-LR cells were established from a lenvatinib-resistant mouse model by stepwise induction with escalating doses of lenvatinib in vitro. In brief, cells isolated from drug‑resistant mouse HCC tissues were first cultured with 1 μM lenvatinib. The concentration was doubled every 2–3 passages until cells grew stably in 20 μM lenvatinib for more than 3 months. Following 2 weeks of culture in drug‑free medium, these cells still maintained a high and stable IC50 to lenvatinib, verifying a stably acquired resistant phenotype.

### Transfection of siRNA, plasmid, and lentivirus

The following reagents were utilized: siRNA targeting ITGA5 (#HX-H-S3-17,044, Cas9X) and RPRD1A (#HX202412237666, Cas9X); overexpression plasmids for c-JUN (#CH836318, WZ Biosciences) and shRNA against RPAP2 (#G64100, GENERAY). Transient transfections employed Lipo8000 transfection reagent (#C0533, Beyotime). For stable overexpression, cells were infected with lentiviruses encoding human RPRD1A (#GOSL0393655, GENE) or empty vector control (#LVCON254, GENE), followed by puromycin selection (4 μg/mL). All nucleic acid sequences appear in Table S6.

### IC50 and CCK8 assay

For IC50 determination, 5 × 10^3^ cells/well were seeded in 96-well plates. Following 24-h adhesion, cells received gradient concentrations of lenvatinib (#S1164, Selleck) (pre-treated with siRNA for 48 h where applicable). After 48-h of drug exposure, viability was assessed using CCK-8 (#A311-02-AA, Vazyme): 90 μL serum-free medium and 10 μL CCK-8 reagent were added per well, incubated at 37 °C for 2 h (light-protected), followed by OD450 measurement. IC50 values were derived using GraphPad Prism nonlinear regression.

For proliferation kinetics, 2 × 10^3^ cells/well were plated. Post-adhesion, cells underwent continuous stimulation with fixed-concentration Drug. At 24-h intervals, medium was replaced with 90 μL serum-free medium and 10 μL CCK-8. OD450 readings were recorded after 2 h incubation (37 °C, dark), and measurements were taken five times in total.

### Cell flow apoptosis assay

Following lenvatinib treatment (48 h, fixed concentration), apoptosis was quantified using flow cytometry. Briefly, 5 × 10^5^ cells/well were plated in 6-well plates. After 24-h adhesion, cells were exposed to lenvatinib. Subsequently, cells were harvested and dually stained with FITC-conjugated Annexin V and propidium iodide (PI) per manufacturer’s protocol (#C1062L, Beyotime). Samples were analyzed within 1 h on the BD FACSCanto II system, collecting ≥ 10,000 events per sample. Viable (Annexin V⁻/PI⁻), early apoptotic (Annexin V⁺/PI⁻), late apoptotic (Annexin V⁺/PI⁺), and necrotic (Annexin V⁻/PI⁺) populations were quantified using FlowJo (v10.8.1) software.

### Western blot

Cellular proteins were extracted using RIPA lysis buffer (#P0013B, Beyotime) containing protease and phosphatase inhibitors (#C0001, #C0002, and #C0003, TargetMol). Lysates were sonicated, centrifuged, and supernatants collected. Protein concentration was determined by BCA assay (#P0010, Beyotime). Samples were mixed with 5 × SDS buffer (#P0015, Beyotime), heat-denatured (95 °C, 5 min), and separated by SDS-PAGE (7.5–12% gels). Proteins were transferred to nitrocellulose membranes (0.2 μm, Amersham). Membranes were blocked with 5% BSA (#ST023, Beyotime) (1 h, RT) then incubated overnight at 4 °C with primary antibodies diluted in Primary Antibody Dilution Buffer solution (#P0023A, Beyotime): β-actin (1:5000; #66,009–1-Ig; Proteintech), IgG isotype control (1:1000; #sc-66931; Santa Cruz), RPRD1A (1:1000; #23,652–1-AP; Proteintech), ITGA5 (1:1000; #ab150361; Abcam), FAK (1:500; #HY-P80125; MCE), p-FAK [Tyr397] (1:1000; #3283S; CST), AKT (1:1000; #9272; CST), p-AKT [Ser473] (1:1000; #9271; CST), ERK (1:1000; #4695; MCE), p-ERK [Thr202/Tyr204] (1:1000; #4370; CST), RPAP2 (1:1000; #17,401–1-AP; Proteintech), RPB1 (1:1000; #14958S; CST), and c-JUN (1:1000; #9165T; CST). Following TBST washes, membranes were incubated (1 h, RT) with fluorescent secondary antibodies (LICOR): Anti-rabbit IRDye 800CW (1:5000; #926–32,211) and Anti-mouse IRDye 680RD (1:5000; #926–32,210). Protein bands were visualized using an Odyssey imaging system (LICOR).

### Immunoprecipitation

Target antibodies were conjugated to Protein A/G magnetic beads (#P2108-5mL, Beyotime) following the manufacturer’s protocol. Bead–antibody complexes were incubated with 500–1000 μg protein lysates overnight at 4 °C with rotation. Beads were then magnetically collected, washed four times with cold IP buffer, and boiled in 1 × SDS buffer at 95 °C for 5 min to elute proteins. Eluted samples were separated by SDS-PAGE and transferred to membranes for western blotting. Rabbit IgG (#sc-66931, Santa Cruz) was used as an isotype control to exclude nonspecific binding.

### Immunohistochemistry

Paraffin-embedded tissues were sectioned at 4 μm, dewaxed in xylene, and rehydrated through a graded ethanol series. Heat retrieval was performed, followed by endogenous peroxidase quenching with 3% H_2_O_2_ for 15 min. After blocking with 1% BSA for 30 min, sections were incubated overnight at 4 °C with primary antibodies. Following washing, horseradish peroxidase-conjugated secondary antibodies (1:50, #A0208 and #A0216, Beyotime) were applied for 1h. DAB chromogen (#K3468, Dako) visualization was performed with hematoxylin counterstaining, after which slides underwent dehydration and sealed.

Quantitative analysis employed a semi-quantitative H-score system evaluated by two blinded pathologists, wherein staining intensity was graded 0 (negative), 1 (pale yellow), 2 (brown-yellow), or 3 (brown-black) while positive cell proportion was scored 1 (< 25%), 2 (25%–49%), or 3 (≥ 50%). Final expression stratification classified specimens as low (H-score 1–3) or high (H-score 4–6) based on the sum of intensity and proportion scores, assessed across five representative fields per section using the microscope.

### Long-term clonogenic assay

Clonogenic survival was assessed by seeding 2 × 10^4^ cells/well in 6-well plates. After 24-h adhesion, cells received drug treatment either immediately or following siRNA transfection. Colonies (> 50 cells/clone) were fixed after 10–14 days with 4% paraformaldehyde (#P0099, Beyotime) for 30 min, then stained with Crystal Violet Staining Solution (#C0121, Beyotime) for quantification. Air-dried plates were digitally imaged and analyzed using Image J (v1.8.0).

### PCR

For gene expression analysis, total RNA was isolated via RNA-Quick Purification Kit (#TB-101, TEYE), reverse-transcribed using HiScript III All-in-one RT SuperMix Perfect for qPCR (#R333-01, Vazyme), and amplified using SYBR® Green (#Q312, Vazyme) on a Roche LightCycler® 480. A three-step amplification protocol was employed for the qPCR reaction. Upon completion of qPCR, the Cq value of each sample well was recorded for subsequent data analysis. Primers used are listed in Table S7.

### Chromatin Immunoprecipitation (ChIP)

Cells were crosslinked with formaldehyde solution (1% final concentration), and the reaction was terminated with glycine solution. Following lysis with SDS Lysis Buffer, chromatin was fragmented by sonication to an average size of 400–800 bp. Protein A/G Magnetic Beads/Salmon Sperm DNA Magnetic Beads were incubated overnight at 4 °C with the chromatin fragments and target primary antibody. Rabbit IgG was used as a negative control. After washing, immunoprecipitated complexes were eluted, crosslinks were broken, and DNA was purified for subsequent PCR analysis. Enrichment of target DNA regions was compared between the target antibody and IgG control groups.

### Transcriptome sequencing

RNA was extracted, and its quality was controlled. When the RNA Integrity Number (RIN) was greater than or equal to 6 and the total RNA amount was greater than or equal to 200 ng, which met the requirements for library construction, the next step could be continued. The standard procedure of Illumina was used for library preparation (using VAHTS Universal V6 RNA-seq Library Prep Kit for Illumina®), and the library quality was assessed. High-throughput sequencing was performed using Illumina NovaSeq 6000. The raw sequencing data were filtered, counted, normalized, and analyzed.

### Phosphoproteome analysis

Protein samples were labeled with biotin. Nonspecific binding sites on the chip were blocked, followed by hybridization with biotinylated samples. After washing, the chip was scanned using an Agilent SureScan Dx Microarray Scanner. Raw signals were extracted with GenePix Pro 6.0. Six technical replicates were included for each antibody on the CSP100 Plus chip. Data were processed using Grubbs’ test to remove outliers, and the mean signal intensity was calculated for each antibody. After mean normalization, the final dataset was used for intergroup comparisons.

### Luciferase reporter gene assay

Cells were first transfected with the c-JUN expression plasmid or empty control plasmid using Lipo8000 Transfection Reagent. Subsequently, cells were co-transfected with firefly luciferase reporter plasmid containing the ITGA5 promoter region and Renilla luciferase control plasmid pRL-TK (#D2760, Beyotime) using Lipo8000. Luciferase activity was measured using the Dual Luciferase Reporter Gene Assay Kit (#11402ES60, Yeason) following the manufacturer’s protocol. Firefly luciferase activity was normalized to Renilla luciferase activity to control for transfection efficiency.

### Animal experiments

All animal procedures strictly adhered to Guidelines for the Management and Use of Laboratory Animals. Male C57BL/6 and BALB/c nude mice (6 weeks) were used for drug resistance, response, and xenograft models. Mice were subcutaneously inoculated with one of the following cell types: 1 × 10^6^ H22 cells, 5 × 10^5^ PLC‑VECTOR cells, 5 × 10^5^ PLC‑LV‑RPRD1A cells, or 1 × 10^6^ H22‑LV‑RPRD1A cells. Upon tumors reaching 50–100 mm^3^ (calculated as 0.5 × length × width^2^), treatments began: (i) Resistance model (n = 6): PBS, single-dose lenvatinib (100 mg/kg, only one time), or maintenance lenvatinib (100 mg/kg, twice weekly); (ii) Xenograft model (n = 6): PBS or lenvatinib (30 mg/kg); (iii) Response model (n = 4): PBS, defactinib (50 mg/kg), volociximab (10 mg/kg), lenvatinib (30 mg/kg), or combinations. Tumors were measured tri-weekly until the 1500 mm^3^ endpoint requiring euthanasia.

### Statistical analysis

Statistical analyses were performed using GraphPad Prism 9 and SPSS 28.0. Differences between two groups were evaluated by unpaired t-test, and one-way ANOVA was used for comparisons among three or more groups. IC50 values were determined via nonlinear regression. Patient survival was estimated using Kaplan–Meier analysis and compared with the log-rank test. A p < 0.05 was regarded as statistically significant.

## Supplementary Information

Supplementary Material 1.

Supplementary Material 2.

Supplementary Material 3.

## Data Availability

All data are available from the corresponding authors upon request.
