# Supplementary material for: RPRD1A drives lenvatinib resistance in hepatocellular carcinoma via the ITGA5-FAK signaling axis
Source: Mol Biomed. 2026 May 22;7:73. doi: 10.1186/s43556-026-00459-8 (PMC13197568; doi:10.1186/s43556-026-00459-8)
Supplement: Supplementary file 1 — Supplementary Material 1. [file 43556_2026_459_MOESM1_ESM.pdf]

RPRD1A Drives Lenvatinib Resistance in Hepatocellular Carcinoma via the ITGA5-FAK Signaling Axis

《Molecular Biomedicine》

Xiaomeng Yao[1,2†], Yunkai Lin[2,3†*], Pingping Chen[2], Mengyou Xu[2], Mengmiao Pei[2], Xinru Fan[1], Huibo Feng[2], Xinhao Xing[2,3], Jiaqi Guo[2,4], Yang Liu[2,4], Xinzhu Quan[2,3], Yufei Pan[2,3], Yexiong Tan[2,3], Huabang Zhou[1], Liwei Dong[2,3,5*], Hui Wang[1*], Heping Hu[1*].

[1] Department of Hepatobiliary Medicine, Eastern Hepatobiliary Surgery Hospital, Second Military Medical University (Naval Medical University), No. 700 Moyu North Road, Jiading, Shanghai 201800, China

[2] National Center for Liver Cancer, Naval Medical University, No. 366 Qianju Road, Jiading, Shanghai, 201800, China

[3] International Cooperation Laboratory on Signal Transduction, Eastern Hepatobiliary Surgery Hospital, Second Military Medical University (Naval Medical University), No. 225 Changhai Road, Yangpu, Shanghai 200438, China

[4] Department of Oncology, Eastern Hepatobiliary Surgery Hospital, Second Military Medical University (Naval Medical University), No. 700 Moyu North Road, Jiading, Shanghai 201800, China

[5] Laboratory of Signaling Regulation and Targeting Therapy of Liver Cancer, the Ministry of Education, No. 225 Changhai Road, Yangpu, Shanghai 200438, China.

[†]Theses authors contributed equally to this work.

*Correspondence:

Yunkai Lin, No. 366 Qianju Road, Jiading, Shanghai, 201800, China, linyunkaikely@163.com

Liwei Dong, No. 366 Qianju Road, Jiading, Shanghai, 201800, China, dlw@smmu.edu.cn

Hui Wang, No. 700 Moyu North Road, Jiading, Shanghai 201800, China, wanghui_ehbh@163.com

Heping Hu, No. 700 Moyu North Road, Jiading, Shanghai 201800, China, huehbh@126.com

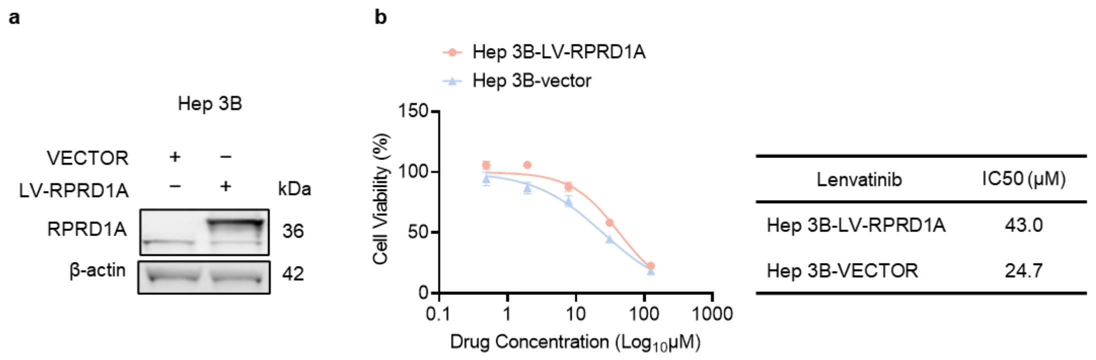

Fig. S1 RPRD1A enhances lenvatinib resistance in HCC.

a. Expression of RPRD1A protein via western blot analysis.

b. Dose-response curves and IC50 values of lenvatinib in Hep3B-LV-RPRD1A and control cells after 48 h treatment (n = 5).

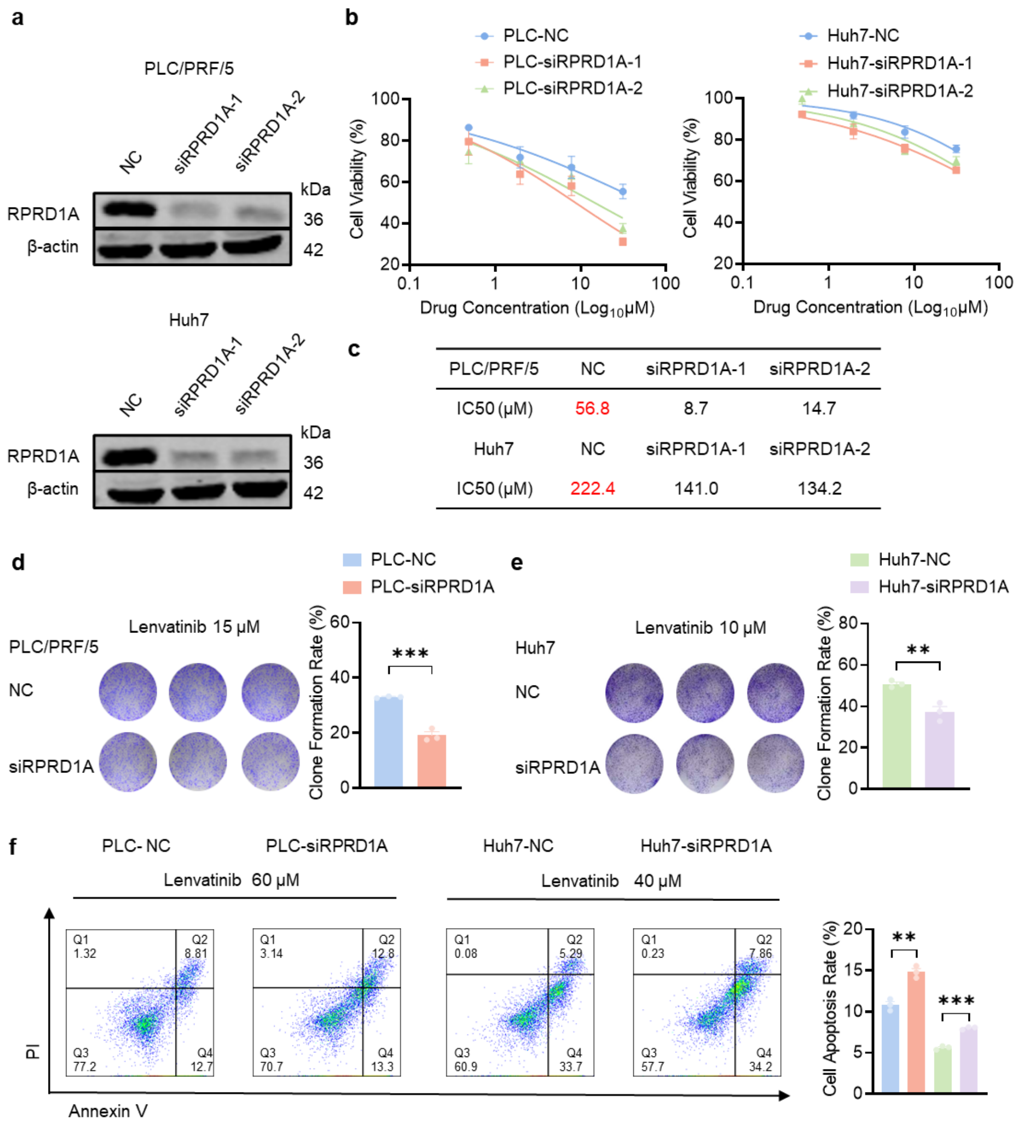

Fig. S2 Knockdown of RPRD1A enhances the sensitivity of HCC to Lenvatinib.

a. Expression of RPRD1A protein in PLC/PRF/5 (upper panel) and Huh7 (lower panel) cells transfected with siRPRD1A via western blot analysis.

b. Dose-response curves of siRPRD1A-transfected PLC/PRF/5 (left panel) and Huh7 (right panel) cells and their control cells treated with Lenvatinib for 48 hours (n = 3).

c. Lenvatinib IC50 values of (b) were calculated based on cell viability.

d-e. Representative images and statistical analysis of long-term colony formation assay of RPRD1A-knockdown PLC/PRF/5 (left panel), Huh7 cells (right panel) and their control cells. Cells were grown in the presence of Lenvatinib at the indicated concentrations for 14 days (n = 3).

f. Flow cytometric quantification of apoptotic cells, identified by Annexin V-FITC/PI staining, in RPRD1A-knockdown PLC/PRF/5 (left panel), Huh7 cells (right panel) and their control cells treated with Lenvatinib at indicated concentrations for 48 hours (n = 3). Data are presented as mean ± SEM, statistical significance: ns. not significant, $**p < 0.01$, $***p < 0.001$.

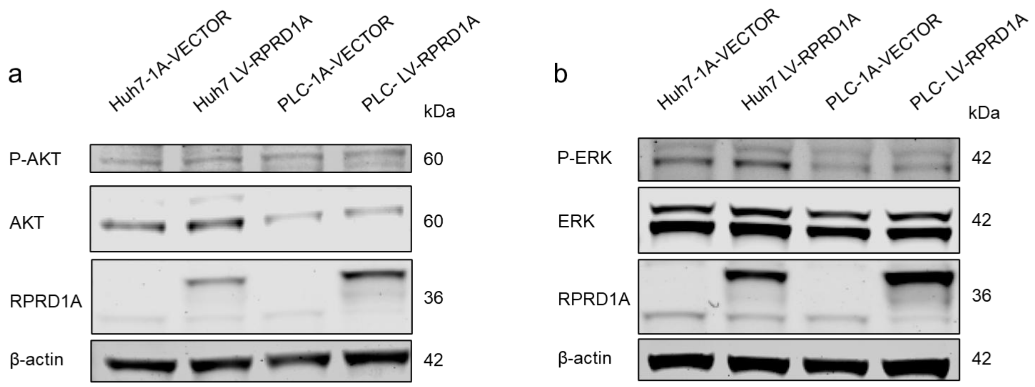

Fig. S3 Overexpression of RPRD1A fails to activate the PI3K-AKT-mTOR or MEK-ERK signaling pathways.

a. Expression of phosphorylated AKT protein (Ser473) in Huh7-LV-RPRD1A and their control cells, PLC-LV-RPRD1A and their control cells via western blot analysis.

b. Expression of phosphorylated ERK protein (Thr202/Tyr204) in Huh7-LV-RPRD1A and their control cells, PLC-LV-RPRD1A and their control cells via western blot analysis.

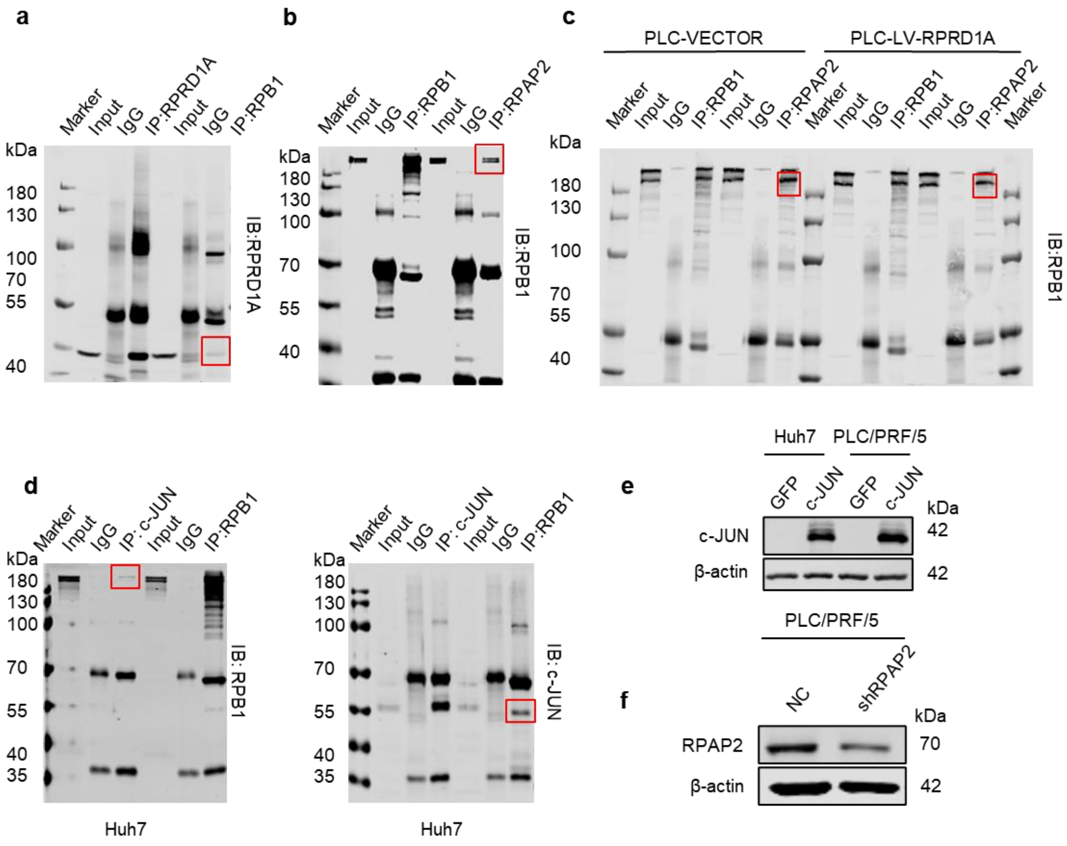

Fig. S4 RRPD1A positively regulates ITGA5 expression by competitively binding RNA pol II with RPAP2 leading to Lenvatinib resistance.

a. Co-IP analysis showing that the RPRD1A protein could bind to RPB1, the largest subunit of RNA pol II in Huh7 cells.

b. Co-IP analysis showing that the RPAP2 protein could bind to RPB1 in Huh7 cells.

c. Co-IP analysis showing attenuated interaction of RPAP2 with RPB1 in PLC-LV-RPRD1A compared to PLC-VECTOR.

d. Co-IP analysis showing that the c-JUN protein can bind to RPB1 in Huh7 cells.

e. Expression of c-JUN protein in c-JUN plasmid-transfected PLC/PRF/5, Huh7 cells and the control cells via western blot analysis.

f. Expression of RPAP2 protein in shRPAP2-transfected PLC/PRF/5 and the control cells via western blot analysis.

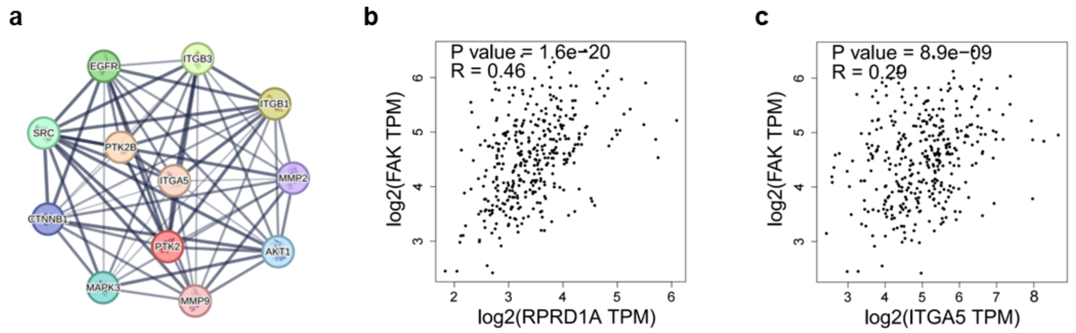

Fig. S5 ITGA5 induces resistance to Lenvatinib in RPRD1A-overexpressing HCC cells through the FAK pathway.

a. PPI network showing the proteins clustered in the FAK pathway, with interactions between ITGA5 and proteins of the FAK signaling pathway.

b. Correlation analysis of RPRD1A and FAK expression levels in HCC based on data from the GEPIA2.0 database.

c. Correlation analysis of ITGA5 and FAK expression levels in HCC based on data from the GEPIA2.0 database.

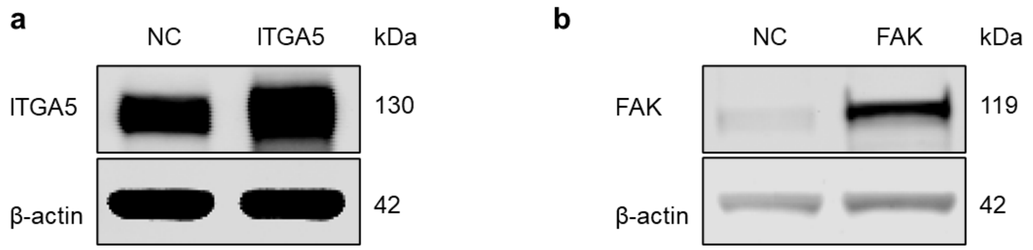

Fig. S6 Validation of ITGA5 and FAK Overexpression efficiencies.

a. Expression of ITGA5 protein in PLC-LV-RPRD1A transfected with ITGA5 plasmid or control constructs via western blot analysis.

b. Expression of FAK protein in PLC-LV-RPRD1A transfected with FAK plasmid or control constructs via western blot analysis.

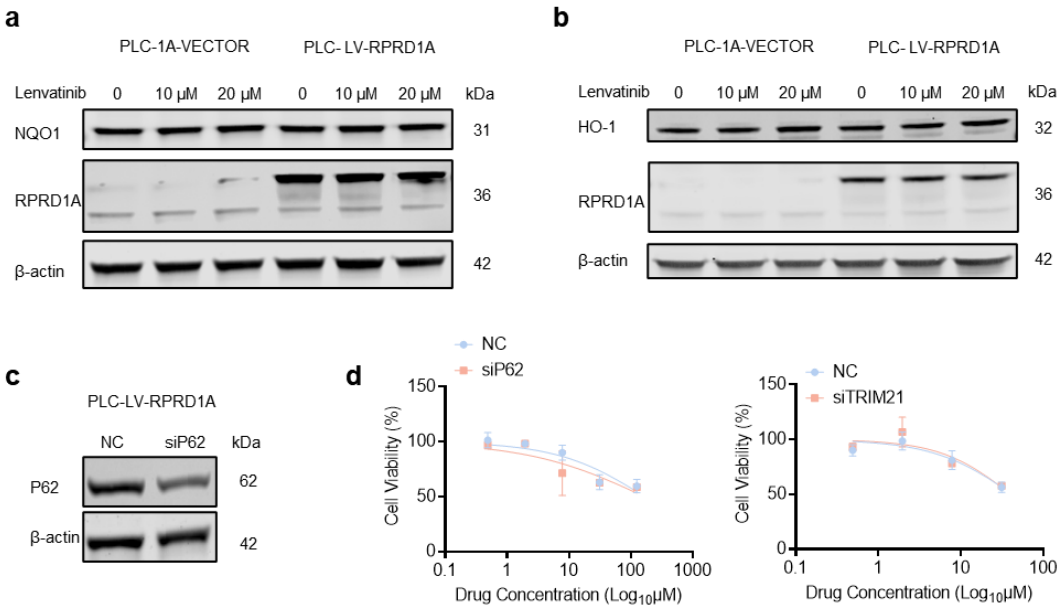

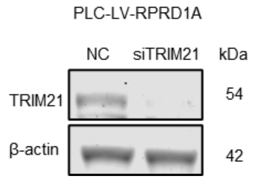

Lenvatinib	IC50 (μM)	Lenvatinib	IC50 (μM)
NC	167.3	NC	42.1
siP62	165.2	siTRIM21	40.1

Fig. S7 RPRD1A Does not Induce Lenvatinib Resistance Via the NRF2 Axis

a. Expression of NQO1 protein in PLC-LV-RPRD1A and the control cells treated with different concentrations of Lenvatinib (0, 10, 20 μM) via western blot analysis.

b. Expression of HO-1 protein in PLC-LV-RPRD1A and the control cells treated with different concentrations of Lenvatinib (0, 10, 20 μM) via western blot analysis.

c. Expression of P62 protein in PLC-LV-RPRD1A (upper panel) and TRIM21 protein in PLC-LV-RPRD1A (lower panel) cells transfected with siRNA via western blot analysis.

d. Dose-response curves of siP62-transfected PLC-LV-RPRD1A (left panel) and siTRIM21-transfected PLC-LV-RPRD1A (right panel) cells and their control cells treated with Lenvatinib for 48 hours (n = 3).

Table S1 Expression Levels of RPRD1A and ITGA5 and Corresponding ORR in HCC Patients Treated with Lenvatinib

RPRD1A	High	Low	ITGA5	High	Low
Cases	19	11	Cases	25	5
ORR (%)	21.1	36.3	ORR (%)	24.0	40.0

Table S2 Multivariate Survival Analysis of Prognostic Factors for Recurrence-Free Survival in Patients with HCC

Characteristics	HR	95% CI	*p* values
ITGA5	4.993	(1.647, 38.542)	0.023
RPRD1A	4.886	(1.538, 15.521)	0.007

Table S3 Definitions of Lenvatinib Sensitivity and Resistance

Group	Definition	Number of patients
Lenvatinib sensitivity	HCC patients with Lenvatinib monotherapy or combined immunotherapy had non-PD (CR/PR/SD) on radiological assessment (RECIST 1.1 criteria), with the non-PD status sustained for ≥ 6 months.	12
Lenvatinib Primary resistance	HCC patients with Lenvatinib monotherapy or combined immunotherapy had PD on radiological assessment (RECIST 1.1 criteria) within 6 months after initial treatment.	13
Lenvatinib acquired resistance	HCC patients with Lenvatinib monotherapy or combined immunotherapy had non-PD (CR/PR/SD) (RECIST 1.1 criteria) on radiological assessment within 6 months after initial treatment, but PD after 6 months.	5

Table S4 Patient Treatment Regimens

Therapy	Number of patients
Lenvatinib monotherapy	24
Lenvatinib + Tislelizumab	1
Lenvatinib + Sintilimab	5

Table S5 Clinicopathological Data of Patients Treated with Lenvatinib

Variables	All patients n = 30)	High-response Group （n = 12）	Low-response Group （n = 18）	χ2 or Z values	*p* values
Age				0.625	0.429
≥ 60 years	10	3（25%）	7（39%）		
< 60 years	20	9（75%）	11（61%）		
Gender				2.520	0.112
Male	23	11（92%）	12（67%）		
Female	7	1（8%）	6（33%）		
Smoking and drinking				1.094	0.296
Yes	14	7（58%）	7（39%）		
No	16	5（42%）	11（61%）		
Hepatitis B				0.106	0.745
Yes	21	8（67%）	13（72%）		
No	9	4（33%）	5（28%）		
Hepatitis C				/	0.490
Yes	1	0（0%）	1（0.1%）		
No	29	12（100%）	17（99.9%）		
Cirrhosis				0.028	0.867
Yes	22	9（75%）	13（72%）		
No	8	3（25%）	5（28%）		
AFP (μg/L)				0.215	0.643
< 20	11	5（42%）	6（33%）		
≥ 20	19	7（58%）	12（67%）		

CA199 (U/mL)				< 0.001	1
≥ 37	5	2（17%）	3（17%）		
< 37	25	10（83%）	15（83%）		
Number of tumors				1.300	0.254
1	21	7（58%）	14（78%）		
≥ 2	9	5（42%）	4（22%）		
Tumor size (cm)				< 0.001	1
≤ 5	15	6（50%）	9（50%）		
> 5	15	6（50%）	9（50%）		
Tumor Location				4.290	0.117
Left Lobe	9	1（17%）	8（44%）		
Right Lobe	15	7（66%）	8（44%）		
Other	6	4（25%）	2（11%）		
MVI				2.790	0.247
0	13	7（58%）	6（33%）		
1	5	2（17%）	3（17%）		
2	12	3（25%）	9（50%）		
Grade				/	1
I-II	1	0（0%）	1（0.1%）		
III-IV	29	12（100%）	17（99.9%）		
TNM				0.096	0.757
I-II	19	8（67%）	11（61%）		
III-IV	11	4（33%）	7（39%）		

Table S6 siRNA, plasmid and shRNA sequences

	Sequence (5'-3')
siITGA5#1	AAACACGUUGCUGACUCCAUUGGUU
siITGA5#2	CCUCAGGAACGAGUCAGAAUUUCGA
siRPRD1A#1	GCAACUCACUCGAAUGUUATT
siRPRD1A#2	CGAACUUAUGAACAGAUAATT
c-JUN	CGCAAATGGGCGGTAGGCGTG
shRPAP2#1	gcaggtactaaacagacaagtctcgagacttgtctgtttagtacctgc
shRPAP2#2	ggcgattgcaaattagatagtCTCGAGactatctaatttgcaatcgcc

Table S7 Primers Used for Quantitative Real-Time PCR (qPCR)

Genes	Sequence (5'-3')
18S-Forward	CGGCTACGACATCCAAGGAA
18S-Reverse	GCTGGAATTAGCGCGGCT
mCRIP1-Forward	AAGTGCGACAAGGAGGTGTAT
mCRIP1-Reverse	AGAGGTCAGTGTCTTTCCACATT
mENTPD3-Forward	TTGTGAGCATTGTGGTACTTGT
mENTPD3-Reverse	GGCCACTGATACACGTAGACAG
mPER1-Forward	TGAAGCAAGACCGGGAGAG
mPER1-Reverse	CACACACGCCGTCACATCA
mSAA3-Forward	TGCCATCATTCTTTGCATCTTGA
mSAA3-Reverse	CCGTGAACTTCTGAACAGCCT
mTPPP3-Forward	AGCGGGCAAGAGATGAATGG
mTPPP3-Reverse	GCAGATTTCGCCTTGACTTTG
mRPRD1A-Forward	CAGCCCTTCTCCTCCCAAA
mRPRD1A-Reverse	CATTCTCACATCTGCAAGTCCT
hITGA5-Forward	GGCTTCAACTTAGACGCGGAG
hITGA5-Reverse	TGGCTGGTATTAGCCTTGGGT
hRPRD1A-Forward	CAGCCCTTCTCCTCCCAAA
hRPRD1A-Reverse	CATTCTCACATCTGCAAGTCCT
